# Not All Stressors Are Equal: Mechanism of Stressors on RPE Cell Degeneration

**DOI:** 10.3389/fcell.2020.591067

**Published:** 2020-11-19

**Authors:** Yao Tong, Shusheng Wang

**Affiliations:** ^1^Department of Cell and Molecular Biology, Tulane University, New Orleans, LA, United States; ^2^Department of Ophthalmology, Tulane University, New Orleans, LA, United States

**Keywords:** AMD, RPE, oxidative stress, senescence, cell death

## Abstract

Age-related macular degeneration (AMD) is a major cause of irreversible blindness among the elderly population. Dysfunction and degeneration of the retinal pigment epithelial (RPE) layer in the retina underscore the pathogenesis of both dry and wet AMD. Advanced age, cigarette smoke and genetic factors have been found to be the prominent risk factors for AMD, which point to an important role for oxidative stress and aging in AMD pathogenesis. However, the mechanisms whereby oxidative stress and aging lead to RPE cell degeneration are still unclear. As cell senescence and cell death are the major outcomes from oxidative stress and aging, here we review the mechanisms of RPE cell senescence and different kinds of cell death, including apoptosis, necroptosis, pyroptosis, ferroptosis, with an aim to clarify how RPE cell degeneration could occur in response to AMD-related stresses, including H_2_O_2_, 4-Hydroxynonenal (4-HNE), N-retinylidene-N-retinyl-ethanolamine (A2E), Alu RNA and amyloid β (Aβ). Besides those, sodium iodate (NaIO_3_) induced RPE cell degeneration is also discussed in this review. Although NaIO_3_ itself is not related to AMD, this line of study would help understand the mechanism of RPE degeneration.

## Age-Related Macular Degeneration

Age-related macular degeneration (AMD) is a major cause of irreversible blindness among the elderly population. The prevalence of AMD is projected to reach 288 million in 2040 due to the increase of the aging population, which could lead to low life quality of the elderly and represent a significant economic burden to the society (Pascolini and Mariotti, 2012; Wong et al., 2014). Macula is the central region of the retina which is critical for the central vision. Early AMD is characterized by drusen deposit underneath the Bruch membrane and disordered pigmentation in the choroid/retinal pigment epithelium (RPE) layers in the macula (de Jong, 2006; Jager et al., 2008). Late AMD has both “dry” and “wet” forms. Geographic atrophy (GA), the advanced form of dry AMD, is featured by the irreversible loss of the RPE, photoreceptors (PRs) and choriocapillaris, which eventually lead to vision loss. Choroidal neovascularization (CNV), as shown by the abnormal growth of new and leaky blood vessels from the choroid into the retina, is a hallmark of wet AMD. Dry and wet AMD accounts for 80–90% and 10–20% of AMD cases, respectively (Bressler, 2002). Currently, antibodies to vascular endothelial growth factor (VEGF) have been approved by FDA to treat wet AMD. Although some clinical studies have shown that antioxidant vitamins and zinc supplements help to slow AMD disease progression (Age-Related Eye Disease Study Research, 2001; Age-Related Eye Disease Study 2 Research, 2013), there is no available cure for dry AMD. The pathogenesis of AMD is still unclear, genetic, environmental factors as well as advanced age, each contributes to the disease progression (Klein et al., 1998; Tuo et al., 2004; Jonasson et al., 2011). Genome-wide association study has identified a list of AMD-associated genetic variations, including Complement factor family members (Edwards et al., 2005; Haines et al., 2005; Klein et al., 2005), Apolipoprotein E (APOE) (Ngai et al., 2011), Fibroblast growth factor 2 (FGF2) (Brion et al., 2011), DNA excision repair protein (ERCC6) (Baas et al., 2010) and Age-related maculopathy susceptibility protein 2 (ARMS2) (Micklisch et al., 2017). However, the No. 1 risk factor for AMD is advanced age, with one third of adults over 75 are affected by AMD (Jonasson et al., 2011). Persons over 85 years old have 10 folds higher prevalence of late AMD than persons who are 70–74 years old (Jonasson et al., 2011). Cigarette smoking, which induces systemic oxidative stress, is the second most consistent and modifiable risk factor for AMD development, associated with 2 to 3 folds increased risk for AMD (Klein et al., 1993).

## Reactive Oxygen Species, Oxidative Stress and AMD

**FIGURE 1:**
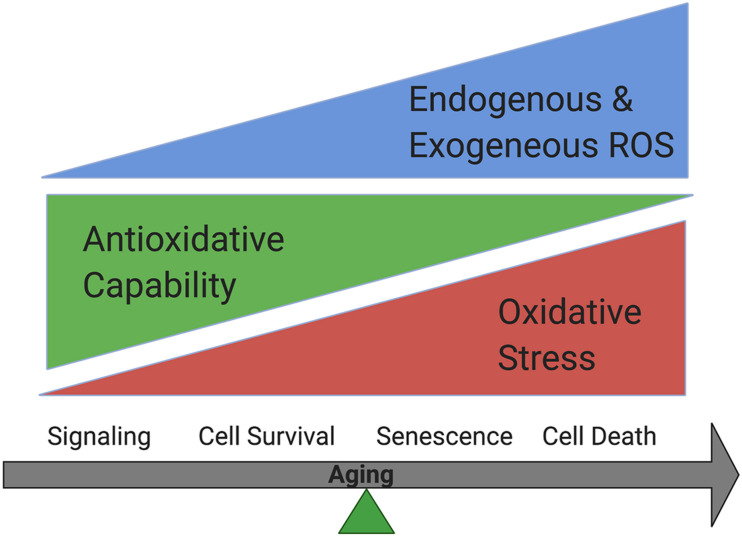
Oxidative stress accumulation increases while antioxidative capability decreases in cells during aging. Cells could survive when oxidative stress is balanced by antioxidative systems while senescence could occur under mild oxidative stress and cell death could happen under more severe oxidative stress.

Free radicals include reactive oxygen species (ROS) and reactive nitrogen species (RNS). They are produced during normal metabolism as well as in some pathological conditions (Phaniendra et al., 2015). ROS regulate cellular homeostasis and could contribute to disease pathophysiology, including AMD (Beatty et al., 2000). In normal cells, ROS are produced during metabolic process by enzymes including nicotinamide adenine dinucleotide phosphate [NADPH] oxidases (Noxes), other oxidases and lipoxygenases, and serve as active regulators of cellular signaling. They are balanced by powerful antioxidative systems. Excessive ROS production could occur when cells are exposed to exogenous oxidative stressors, including UV light, ionizing radiation, diet and cigarette smoking. Oxidative stress accumulation due to the increased endogenous and exogenous ROS, and/or decreased antioxidative capability could lead to oxidative modification to major cellular macromolecules, which lead to features of aging including metabolic dysfunction, cell senescence or cell death (Beckman and Ames, 1998; Droge, 2002; Valko et al., 2007; Rajendran et al., 2014; Davalli et al., 2016; Pizzino et al., 2017; Figure 1).

Reactive oxygen species consist of Superoxide radicals (O_2_^⋅–^), hydrogen peroxide (H_2_O_2_), hydroxyl radicals (OH^⋅^), and singlet oxygen (^1^O_2_). Molecular oxygen (O_2_) undergo single electron reduction and form superoxide anion (O_2_^⋅–^). Once formed, O_2_^⋅–^ is involved in a reaction that in turn generates H_2_O_2_ (2O_2_^⋅–^ + 2H^+^ → H_2_O_2_ + O_2_). Hydroxyl radical (OH^⋅^) is generated by reaction of O_2_^⋅–^ with H_2_O_2_ through Fenton reaction (Fe^2+^ + H_2_O_2_ → Fe^3+^ + OH^⋅^ + OH^–^) (Winkler et al., 1999). Peroxynitrite (ONOO^–^) is also a physiological product generated by the interaction of superoxide (O_2_^⋅–^) and nitric oxide (NO) which can generate ROS and induce cell death (Szabo et al., 2007). High intracellular ROS level could modify and damage carbohydrates, membrane lipids, proteins, and nucleic acids, with pathological consequences. ROS can easily react with membrane lipids and cause the lipid peroxidation. Polyunsaturated fatty acids (PUFAs) are particularly susceptible to free radical damage which generates products including malondialdehyde (MDA) and 4-hydroxynonenal (4-HNE) which show higher levels in AMD retina (Gardner, 1989; Halliwell and Chirico, 1993; Spiteller et al., 2001). Oxidation of docosahexaenoate (DHA)-containing lipids produces carboxyethylpyrrole (CEP), which is also abundant in AMD retina (Crabb et al., 2002). Oxidation in proteins can cause fragmentation, cross-linking, aggregation of proteins, and enhanced proteolysis (Negre-Salvayre et al., 2008). For example, reactive carbonyl compounds formed during lipid peroxidation, such as MDA and 4-HNE, form adducts and cross-links with proteins, which causes protein damage and functional deficiency (Stadtman and Levine, 2000; Negre-Salvayre et al., 2008). ROS-induced nuclear and mitochondrial genomes damage, such as DNA strand breaks, base modifications and DNA-protein cross linkages, are associated with aging and age-related diseases (Bohr et al., 1998, 2007).

Antioxidative systems, include enzymatic and non-enzymatic systems, have evolved to protect against ROS. Enzymatic antioxidants include superoxide dismutase (SOD) and glutathione peroxidase (GPX) et al. SOD catalyze superoxide converse to H_2_O_2_ and O_2_ and reduce ROS levels (Halliwell and Gutteridge, 1986). GPX reduces H_2_O_2_ and lipid peroxides to water and lipid alcohols via the expense of reduced glutathione (GSH) (Arthur, 2000). Thus, GSH is also considered as a kind of non-enzymatic antioxidant. Other non-enzymatic antioxidants include tocopherol homologs, carotenoids, flavonoids, etc. When accumulated free radicals cannot be eliminated by antioxidant systems, damage to DNA, proteins, and lipids happens, which can subsequently cause cell death and diseases (McCord, 2000; Therond, 2006; Birben et al., 2012).

Reactive oxygen species and oxidative stress both have been implicated in AMD. As mentioned above, cigarette smoking is the #2 risk factor for AMD. Retina is a tissue which continually exposes to light, contains high levels of PUFAs and consumes oxygen at a high rate which all increase ROS production in the cells (Beatty et al., 2000; Khandhadia and Lotery, 2010). It has been reported that increased oxidative DNA damage, as well as the accumulation of CEP, 4-HNE, and MDA, is found with aging in retina tissue (Jarrett and Boulton, 2012). These oxidative products have been shown to induce inflammatory response and retinal phenotype in animal models of AMD (Suzuki et al., 2007; Hollyfield et al., 2008). For more information of oxidative stress and AMD, refer to reviews of Jarrett and Boulton (2012) and Mettu et al. (2012).

## RPE Biological Functions

The RPE monolayer of the retina functions as the outer blood-retina barrier and help to transport nutrients and waste between PRs and choroid. RPE cells in the adults are post-mitotic and polarized with proteins/organelles distributed and/or secreted asymmetrically in apical or basolateral domains of the cells (Burke, 2008). The functions of RPE cells include: (1) Maintaining essential function of the retina. Melanins are synthesized and stored in the melanosome of RPE cells which help to absorb light that pass through the PR layer and also absorb reflected light that may degrade the visual image (Weiter et al., 1986). Melanin synthesis decreases with age (Simon et al., 2008). Some transporters on the membrane of RPE help to provide a stable environment for RPE and nearby cells. For example, sodium/potassium adenosine triphosphatase (Na^+^/K^+^-ATPase) is located apically in RPE cells and helps to maintain the volume, ion concentrations and chemical composition of the subretinal space. These are essential for the functions of neural retina and RPE (Wimmers et al., 2007). (2) Maintaining PRs function. Microvilli of RPE cells envelop and interact with the outer segments (OS) of both rod and cone PRs. PRs regeneration of the PR outer segment (POS) occurs every 7–12 days through phagocytosis function of RPE (Young and Droz, 1968), which protects PRs from chronic oxidative stress exposure (Bok, 1985). (3) Participating in the visual cycle. Visual cycle is the process that cycles retinoids between the rod OS and the RPE. Light isomerizes 11-*cis* retinal into all-*trans* retinal, which is released from the visual pigment opsins, causing visual pigment activation. The photoproducts then enter the RPE, where 11-*cis* retinal is regenerated before returning to PRs (Bernstein et al., 1987). (4) Regulating retinal immune response. RPE cells secret cytokines such as IL-1α, IL-1β, IL-7, TNF-α, IFN-γ, TGF-β. Cytokines secreted by RPE play an important role in the homeostasis of the retina, as well as in inflammatory responses by activation of resident cells and attraction and activation of inflammatory cells (Holtkamp et al., 2001). Overall, RPE cells are critical for metabolism and homeostasis of retina, especially PRs. Due to their exposure to high light and oxygen, oxidized POS and PUFAs, RPE cells are exposed to high oxidative stress conditions, and vulnerable to degeneration if the antioxidative defense mechanism is compromised. Several AMD-related risk factors can affect RPE structure and function. Aging leads to RPE structural changes, such as loss of melanin granules, accumulation of residual bodies, drusen formation, thickening of Bruch’s membrane, RPE microvilli atrophy and et al. (Bonilha, 2008). Also, factors such as cigarette smoking, high fat diet and genetic factors are believed to lead to oxidative stress and inflammation which are related to RPE degeneration (Datta et al., 2017). For more information regarding RPE function, refer to review of Sparrow et al. (2010).

## Modes of Cell Degeneration and Death

**FIGURE 2:**
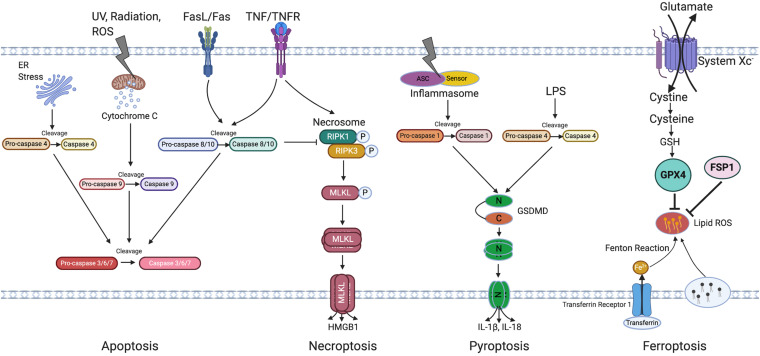
Overview of cell death pathways. Apoptosis is triggered by either intrinsic (by UV, radiation, exogenous ROS, or ER stress) or extrinsic signals (through FasL/Fas or TNF/TNFR) and is regulated by the caspase family of proteins. Caspase 4, 9, and 8/10 serve as initiator caspase, while Caspase 3/6/7 serve as executor caspase. Necroptosis is initiated by activation of TNFR and subsequent activation of necrosome containing phosphorylated RIPK1 and RIPK3, phosphorylation and oligomerization of MLKL then result in cell rupture, death and HMGB1 release. Pyroptosis is induced through both Caspase-1 and Caspase-4 activation which induce GSDMD cleavage, followed by oligomerization of N terminal of GSDMD which then leads to cell rupture and death. Caspase 1 cleaves IL-1β and IL-18, leading to its activation and release from the cells. Ferroptosis is induced by the inhibition of system Xc and is featured by accumulation of lipid ROS which can be inhibited by GPX4 and FSP1. Irons are transferred through transferrin and its receptor and are involved in the Fenton reaction leading to lipid peroxidation. ER, Endoplasmic reticulum; UV, Ultraviolet light; TNF/TNFR, Tumor necrosis factor/Tumor necrosis factor receptor; RIPK3, Receptor-interacting protein kinase 3; MLKL, Mixed Lineage Kinase Domain Like Pseudokinase; HMGB1, High mobility group box 1; ASC, Apoptosis-associated speck-like protein containing a caspase recruitment domain; LPS, Lipopolysaccharide; N-GSDMD, N terminal of gasdermin D; C-GSDMD, C terminal of gasdermin D; IL-1β, Interleukin-1β; IL-18, Interleukin-18; GSH, Glutathione; GPX4, Glutathione Peroxidase 4; FSP1, Ferroptosis suppressor protein 1; ROS, Reactive oxygen species.

Retinal pigment epithelial degeneration in AMD involves RPE dysfunction, senescence and cell death. This review will focus on RPE senescence and cell death. An overview of cellular senescence and cell death mechanisms will first be introduced (Figure 2 Overview of cell death pathways).

### Apoptosis

Apoptosis is a classic mode of programmed cell death. It plays important roles in both physiological and pathological processes. Classic features of apoptosis include membrane blebbing, cell shrinkage, nuclear fragmentation, chromatin fragmentation and the formation of apoptotic bodies (Kerr et al., 1972). Apoptosis can be initiated by either intrinsic or extrinsic pathways. The intrinsic apoptosis pathway is promoted by cellular stresses include DNA damage, oxidative stress and irradiation. The extrinsic pathway relies on signaling through transmembrane receptors of the tumor necrosis factor (TNF) receptor family (Igney and Krammer, 2002). Apoptosis is regulated by the caspase family of proteins. Caspases are synthesized as inactive proenzymes containing a N-terminal peptide or pro-domain, and two subunits. Cleavage of caspases occurs at specific asparagine (Asn) residues located after the pro-domain and between the large and small subunits, forming active heterotetramers. Caspase-8/10 act as initiator caspases which are activated by extrinsic signal. Caspase-9 also functions as initiator caspase but is activated by intrinsic signal. Endoplasmic reticulum (ER) stress could induce the activation of Caspase-4 (Hitomi et al., 2004). These signals then activate downstream caspases 3/6/7 and subsequent apoptosis (Cohen, 1997). Caspases-3/6/7 are considered as executioner caspases due to their similar short pro-domains. Caspase-3 is needed for efficient cell death and also could block ROS production, but activation of Caspase-6 alone cannot cause apoptosis (Gray et al., 2010). Caspase-7 is responsible for ROS production and aids in cell detachment during apoptosis (Brentnall et al., 2013). Usually, active Caspase-3 is detected in most apoptotic cells. A pan-caspase inhibitor z-VAD-FMK can be used to inhibit apoptosis. For details about apoptosis, refer to review of Elmore (2007).

### Necrosis

Necrosis was considered to be a passive and unregulated cell death in responsive to infections, toxins or trauma. Recent studies showed that some necrosis could be regulated. Regulated necrosis includes but is not limited to necroptosis, pyroptosis and ferroptosis.

#### Necroptosis

Necroptosis is morphologically characterized by cells swelling and bursting, with releasing their intracellular contents. It can be initiated by activation of TNF receptor (TNFR) and subsequent activation of two members of the receptor interacting protein kinase (RIPK) family (RIPK1 and RIPK3), when Caspase-8 is not activated (Fritsch et al., 2019). These kinases then form “necrosomes” via specialized domains termed RIP homotypic interaction motifs (RHIM). Reciprocal interactions between RIPK1 and RIPK3 lead to phosphorylation of a pseudokinase called Mixed lineage kinase domain like pseudokinase (MLKL) (Rodriguez et al., 2016). Once phosphorylated, MLKL translocates to the cell membrane and form tetramers, leading to osmotic cell membrane rupture by disrupting cellular ion homeostasis and the release of inflammatory cytokines such as high-mobility group box-1 (HMGB1) (Sun et al., 2012; Dondelinger et al., 2014; Gong et al., 2017). Several inhibitors can be used to block necroptosis, include Necrostatin-1 (Nec-1), a direct RIPK1 inhibitor; Necrostatin-5 (Nec-5), an indirect RIPK1 inhibitor; Necrostatin-7 (Nec-7) that targets RIPK1-independent necrosis; GSK’872, a specific RIPK3 inhibitor and Necrosulfonamide (NSA), a MLKL inhibitor. For details about necroptosis, refer to review of Weinlich et al. (2017).

#### Pyroptosis

Pyroptosis is featured by plasma membrane rupture and release of proinflammatory intracellular contents, include Interleukin-1 beta (IL-1β) and Interleukin-18 (IL-18) (He et al., 2015). Pyroptosis can be induced through both canonical and non-canonical inflammasome pathways (Liu and Lieberman, 2017; Xu et al., 2018). In canonical pyroptosis, inflammasomes include absent in melanoma 2 (AIM2), Pyrin, or the nucleotide-binding oligomerization domain (NOD)-like receptor (NLR) family (NLRP1, NLRP3, and NLRC4) are activated by pathogen-associated molecular patterns (PAMPs) or danger-associated molecular patterns (DAMPs) (Wang et al., 2019). Inflammasomes then recruit Caspase-1 via the CARD-domain containing adaptor protein (ASC, also called PYCARD), which cleave pro-Caspase-1 to its active form. The activated Caspase-1 subsequently induces the maturation and secretion of IL-1β and IL-18 and cleaves gasdermin D (GSDMD) into N-terminal and C-terminal domains. The N-terminal fragments then oligomerize, translocate to the cell membrane and form membrane pores which leads to cell swelling, membrane rupture, release of inflammatory factors and cell death (Ding et al., 2016; Liu et al., 2016). In the non-canonical pathway, Caspase-4/5 (Caspase-11 in mice) recognize cytosolic lipopolysaccharide (LPS) via CARD domain and subsequent GSDMD cleavage which then leads to cell death (Kayagaki et al., 2011; Shi et al., 2014; Kovacs and Miao, 2017). Inhibitors of pyroptosis include Ac-YVAD-CMK, a caspase-1 inhibitor; MCC950, a NLRP3 inhibitor and so on. For details about pyroptosis, refer to review of Bergsbaken et al. (2009).

#### Ferroptosis

Ferroptosis is a regulated cell death defined in 2012 (Dixon et al., 2012) and is characterized by lipid peroxidation and iron involvement, but its molecular pathway is yet to be clearly defined. Ferroptotic cells do not show the typical morphological characteristics of necrosis, such as cell swelling and cell membrane rupture, but mainly display mitochondria shrinkage, increased mitochondria membrane density and mitochondrial cristae reduction (Yagoda et al., 2007; Yang and Stockwell, 2008). It can be induced by the inhibition of system X^c–^, a glutamate/cystine antiporter on the cell membrane. System X^c–^ helps cells to take up cysteine, which stimulates the synthesis of GSH. This promotes the activity of glutathione peroxidase 4 (GPX4), an antioxidative enzyme which reduces lipid hydroperoxides and lipid ROS production in cells (Brigelius-Flohe and Maiorino, 2013). Inhibition of System X^c–^ or GPX4 activity leads to lipid ROS accumulation. Extreme accumulation of lipid ROS is toxic to the cells and results in ferroptosis. It’s been reported that FSP1/AIFM2 functions to suppress ferroptosis, representing a new pathway to regulate ferroptosis (Bersuker et al., 2019; Doll et al., 2019). Lipid ROS scavenger, Liproxststatin-1, Ferrostatin-1, Vitamin E can block ferroptosis. Iron chelator DFO can be used to inhibit ferroptosis as well. For details about ferroptosis, refer to review of Li et al. (2020).

### Cellular Senescence

Cellular senescence was first identified as a stable cell cycle exit from cell culture (Hayflick, 1965). It is now considered as a protective stress response, which includes metabolic reprogramming, chromatin rearrangement and autophagy modulation (Kuilman et al., 2010). Senescent cell accumulation could drive aging and age-related diseases (van Deursen, 2014; Childs et al., 2015). Senescent cells show enlarged cell size, arrested growth, increased ROS levels, persistent DNA damage response, apoptosis resistance, changes in chromatin organization and gene expression (Ogryzko et al., 1996; Chen et al., 2000; Hampel et al., 2004; Herbig et al., 2004). Various biomolecules also can be released by senescent cells to exert changes to neighboring cells, including chemokines, cytokines, proteases, growth factors, which is called senescence-associated secretory phenotype (SASP) (Nelson et al., 2012). Senescence associated (SA)-β-gal can be detected in most senescent cells and acts a marker for senescence (Dimri et al., 1995; Lee et al., 2006). Other upregulated markers for senescence include cell cycle regulators p16^INK4a^, p21, and p53 (Collado and Serrano, 2010). Rapamycin and related mTORC1 inhibitors, ruxolitinib, glucocorticoids and metformin can be used to inhibit senescence. For details about senescence, refer to review of Khosla et al. (2020).

Generally, apoptosis and regulated necrosis are different both morphologically and molecularly. However, cross talk exists among those pathways. For example, inflammasomes mainly mediate pyroptosis, but also can activate Caspase-8 and induce apoptosis (Hitomi et al., 2004; Liu and Lieberman, 2017). Also, NLRP3 inflammasome can be activated by RIPK3 and MLKL which then leads to IL-1β inflammatory responses (Kang et al., 2013). In mouse erythroid precursors, GPX4 which is a key antioxidant in ferroptosis pathway was found to also prevent necroptosis (Canli et al., 2016). In some conditions, cells could go through alternative pathway. Once TNFR is activated, apoptosis happens when Caspase-8 exists but necroptosis could be induced in the absence of Caspase-8 in cells (Fritsch et al., 2019). Ferroptosis and necroptosis were also found to be alternative pathways since deletion of MLKL block necroptosis and cells will go through ferroptosis (Muller et al., 2017). Different modes of cell death have been reported in RPE cells, depending on the type, dosage and duration of stresses. This creates some controversy regarding how RPE cells die response to different stresses in AMD. A clear answer to this question could facilitate therapeutic development for dry AMD, especially GA. Thus, we review here the recent understanding of RPE cell senescence and death mechanism in response to AMD-relevant stresses, including H_2_O_2_, 4-HNE, N-retinylidene-N-retinyl-ethanolamine (A2E), Alu RNA and Amyloid beta (Aβ). Besides those, sodium iodate (NaIO_3_) induced RPE cell degeneration is also discussed in this review. Although NaIO_3_ itself is not related to AMD, this line of study would help understand the mechanism of RPE degeneration.

## Different Stresses in RPE Senescence and/or Cell Death

### Hydrogen Peroxide (H_2_O_2_)

Hydrogen peroxide functions during both normal metabolism and under oxidative stress conditions (Sies and Chance, 1970). The sources of H_2_O_2_ include one or two-electron reduction reactions catalyzed by Noxes and other oxidases (Bedard and Krause, 2007), as well as the complexes in the mitochondrial respiratory chain (Brand, 2016). When not being metabolized, H_2_O_2_ can convert to OH^⋅^ via the Fenton reaction which increases oxidative damage to the cell. It’s been reported that prolong treatment of RPE cells with low concentration H_2_O_2_ can lead to RPE senescence. Marazita et al. (2016) found that 80% of the ARPE-19 cells exposed to 150 uM H_2_O_2_ and cultured in maintenance medium for 10 days exhibited SA-β-Gal positivity and increased p16^INK4a^ and p21 expression. Higher concentration H_2_O_2_ can lead to cell death and the mode of cell death induced by H_2_O_2_ appears to be dependent on its concentration. Many studies reported apoptosis is involved in H_2_O_2_ induced RPE cell death. In Barak’s study, terminal deoxynucleotidyl transferase dUTP nick end labeling (TUNEL) assays combine propidium Iodide (PI)/Annexin V staining were used to detect RPE apoptosis/necrosis after exposure to H_2_O_2_ (0.5–2.5 mM) for 16–24 h. They concluded that H_2_O_2_ at 1 mM induced mostly apoptosis and at 2.5 mM induces mostly necrosis in ARPE-19 cells (Barak et al., 2001). Alge et al. (2002) found increased Caspase-3 activity in H_2_O_2_ treated human RPE cells, as determined by the chromophore p-nitroaniline (pNA) release after its cleavage by activated Caspase-3 from the labeled caspase-specific substrate. Zhao et al. (2019) reported increased Caspase-3 cleavage in H_2_O_2_ treated ARPE-19 cells by Western blotting. There are also many other studies showed apoptosis in H_2_O_2_-treated RPE cells while some chemicals or proteins can protect cells from apoptosis, such as taxifolin (Xie et al., 2017), kinsenoside (Luo et al., 2018), kaempferol (Sreekumar et al., 2005), and genipin (Zhao et al., 2019). Kim et al. (2003) proposed that H_2_O_2_ induced both apoptosis and necrosis in RPE. H_2_O_2_ at 400 uM was shown to induce early apoptosis in ARPE-19 cells with condensed and fragmented nuclei. Higher H_2_O_2_ concentrations lead to late apoptotic and necrotic RPE cell death, while concentrations above 700 uM mainly caused necrotic RPE cell death. Chromatin condensation and marginalization were shown in ARPE-19 cells treated with 500 uM H_2_O_2_ under transmission electron microscope, while 600 uM H_2_O_2_ induces organelle swelling and membrane rupture in the cells. However, in the study of Li et al. (2010), H_2_O_2_ at 400 uM was able to induce ARPE-19 cell death and the cells showed cell swelling, cell membrane rupture, and nuclei condensation which are typical features of necrosis. Our laboratory also studies the nature of oxidative stress induced RPE cell death. In our study, typical necrotic characteristics like PI membrane permeability, RIPK3 activation and HMGB1 release from the nucleus were shown in ARPE-19 cells treated by 300 uM H_2_O_2_. Caspase inhibitor z-VAD could not reduce H_2_O_2_ induced ARPE-19 cell death. However, RIPK1 inhibition and RIPK3 knockdown significantly rescued ARPE-19 cells from H_2_O_2_ treatment (Hanus et al., 2013). One of the major differences between apoptosis and necrosis is the level of cellular ATP. ATP is required for several processes in apoptosis, including caspase activation, enzymatic hydrolysis of macromolecules, chromatin condensation, bleb formation and apoptotic body formation (Richter et al., 1996). Usually, intracellular ATP levels remain unchanged during the whole apoptotic process while ATP depletion happens in necrosis (Eguchi et al., 1997). We found about 90 and 97% ATP depletion in ARPE-19 cells induced by 300 and 500 uM H_2_O_2_ treatment, respectively (Hanus et al., 2013). DNA fragmentation or Caspase-3 cleavage was not seen in H_2_O_2_ treated RPE cells. Some other studies also reported ATP depletion in H_2_O_2_ treated RPE cells (Giddabasappa et al., 2010; Du et al., 2016). It has been found that in human RPE cells, Caspase-8 mRNA and protein levels were low compared with other cell types. Low Caspase-8 levels may protect RPE cells from apoptosis (Yang et al., 2007). Thus, the role of apoptosis in RPE cell death still needs to be verified. So far, there is no report about H_2_O_2_-induced ferroptosis in RPE cells, but a similar stress tert-butyl hydroperoxide (tBH)-induced ferroptosis in ARPE-19 cells has been reported (Marazita et al., 2016). More experiments are still needed to clarify the mechanism of RPE cell death induced by H_2_O_2_. The choice of RPE cells [ARPE-19, primary RPE cells or induced pluripotent stem (IPS)-derived RPE cells] and culture conditions (including cell density, differentiation status) should be carefully controlled.

### 4-Hydroxynonenal (4-HNE)

Lipid peroxidation is the process that oxidants such as free radicals attack lipids containing carbon-carbon double bonds on the membranes of cells and/or subcellular organelles (Yin et al., 2011). 4-HNE is one of the end products of lipid peroxidation. Under physiologic conditions, 4-HNE usually presents at very low concentration in plasma (0.28–0.68 μM) and a bit higher (≤5 μM) in cells under physiologic conditions (Niki, 2009; Schaur et al., 2015). However, its concentration can be increased by 100 times in response to oxidative stress (Esterbauer et al., 1991). 4-HNE concentrations also increase during aging. It was reported in one study that plasma 4-HNE concentration was 68.9 ± 15.0 nmol/L in the young group (up to 30 yr old) which increased to 107.4 ± 27.3 nmol/L in the elderly group (older than 70 yr) (Gil et al., 2006). 4-HNE has protective functions as a signaling molecule at the physiological level but has cytotoxic effect at abnormally high levels (Ayala et al., 2014). 4-HNE accumulation is associated with cell cycle arrest, cell differentiation and cell death (Esterbauer et al., 1991; Niki, 2009; Shoeb et al., 2014). It can also modify histidine, cysteine, and lysine residues of proteins and form HNE-protein adducts. 4-HNE accumulation has been reported to be involved in the pathology of many age-related diseases including Alzheimer’s disease (Skoumalova and Hort, 2012), Parkinson’s disease (Kilinc et al., 1988), and cancer (Nair et al., 2007). 4-HNE has also been shown to be significantly increased in the retina of AMD eyes as well as in patient plasma (Schutt et al., 2003; Ethen et al., 2007). Ethen et al identified nineteen proteins in AMD retina which are involved in energy production and stress response, were consistently modified by 4-HNE regardless of stage of AMD or retinal region (Ethen et al., 2007). Sharma et al reported that 4-HNE induces activation, phosphorylation, and increased nuclear accumulation of p53 in human RPE and ARPE-19 cells. Signaling components involved in p53-mediated apoptosis were activated as well. JNK and Caspase-3 as markers of apoptosis pathway are both activated by 4-HNE as well (Sharma et al., 2008). It’s been found that increased p21 expression (as a senescence marker) was induced by 4-HNE in neuroblastoma cells (Laurora et al., 2005) and colon cancer cells (Cerbone et al., 2007). However, whether 4-HNE could induce senescence in RPE cells hasn’t been reported so far. Based on the definition of ferroptosis, lipid peroxidation is believed to be involved in ferroptosis process. As one of the end products of lipid peroxidation, 4-HNE accumulation was shown in ferroptotic murine heart and kidney tissues (Martin-Sanchez et al., 2017; Fang et al., 2019). It would be interesting to know whether 4-HNE is involved in ferroptosis in RPE cells.

### N-Retinylidene-N-Retinyl-Ethanolamine (A2E)

N-retinylidene-N-retinyl-ethanolamine is a by-product of visual cycle which is formed by the reaction of two *trans*-retinal molecules with phosphatidylethanolamine (Sparrow et al., 2003a). It is a major fluorophore identified in lipofuscin from aged human eyes and is accumulated in RPE with age (Sparrow and Boulton, 2005). A2E undergoes photooxidation and produces oxygen adducts in the presence of blue light and oxygen (Wielgus et al., 2010), which subsequently induces increased oxidative stress and proteins and DNA damage in RPE cells (Ferrington et al., 2016; Sparrow et al., 2003b). Apoptosis has been implicated in A2E accumulated RPE cells in several studies. Shaban et al. (2001) reported A2E induced apoptosis in RPE cells. The cells showed declined mitochondrial activity and release of cytochrome c and apoptosis-inducing factor. They then reported A2E leads to more severe apoptotic cell death in cultured human RPE cells in the light compared to those in the dark (shown by PI/Annexin-V staining). Also, A2E induces increased H_2_O_2_ level and decreased GSH level (Shaban et al., 2002). Sparrow and Cai (2001) observed Caspase-3 activation (using a Caspase-3 fluorescence probe) in ARPE-19 treated with A2E and blue light while an apoptosis inhibitor Z-DEVD-fmk decreased the numbers of apoptotic cells. Alaimo et al. (2019) reported blue light and A2E co-treatment induces ROS generation and increased pro-caspase-3 expression level in ARPE-19 cells. Early and late apoptotic ARPE-19 cells were observed after the treatment. Anderson et al found that A2E induces upregulated IL-1β production and ASC cluster formation in ARPE-19 cells. NLRP3 knock down and Caspase-1 inhibitor Z-WEHD-FMK both can inhibit A2E induced IL-1β production (Anderson et al., 2013). These are makers of pyroptosis which may indicate the involvement of pyroptosis in the process. Wang et al reported that A2E triggers telomere dysfunction and accelerates cellular senescence in ARPE-19 cells (Wang et al., 2018). They found SA-β-gal positivity and SASP in A2E treated ARPE-19 cells, while telomerase overexpression suppressed A2E mediated RPE cell senescence. More studies are needed to clarify the involvement of apoptosis and pyroptosis in A2E-induced RPE degeneration.

### Alu RNA

Alu RNAs are non-coding transcripts belonging to the Alu family of retrotransposons (Cordaux and Batzer, 2009). Free Alu transcripts are expressed at very low levels in physiological conditions, approximately only 10^2^–10^3^ molecules per cell; while the levels increases under various stresses, such as heat shock (Pandey et al., 2011), hyperglycemia (Wang et al., 2016) and viral infection (Panning and Smiley, 1994). It’s been reported that Alu RNA accumulation induces ROS production and impedes SOD2 expression in cells while higher oxidative stress leads to more severe Alu RNA accumulation (Wang et al., 2016; Hwang et al., 2019). Kaneko et al. (2011) found that deficiency of *Dicer1*, an RNase III involved in microRNA biogenesis (Bernstein et al., 2001), leads to cytotoxic Alu RNA accumulation in human RPE cells and RPE degeneration in mice shown by Fundus examination and RPE/choroid flat mount. They showed a more than 40 folds increase in the Alu RNA levels in the RPE of human eyes with GA but not in the neural retina. Also, Dicer1 knockdown in human RPE cells leads to increased Alu RNA accumulation. Subretinal injection of Alu RNA induced RPE degeneration in wild-type mice. Caspase-3 activation was shown in the RPE cells of *Dicer1*^–/–^ mice and Alu RNA-treated human RPE cells. These suggest Dicer1 dysregulation induces Alu RNA accumulation and may cause apoptotic RPE cell death in GA. Tarallo et al. (2012) from the same group showed that Alu RNA exposure induces mitochondria ROS production, activates NLRP3 inflammasome and triggers IL-18 secretion in RPE cells. Inhibition of NLRP3, PYCARD, Caspase-1, or IL-18 alleviates RPE degeneration induced by Dicer1 deletion or Alu RNA exposure. They also observed elevated NLRP3, PYCARD, IL-18 levels and increased Caspase-1 activation in the RPE of human eyes with GA. They showed Alu RNA led to Caspase-1 activation in human RPE cells using both Western blot and a fluorescent reporter of substrate cleavage. Intravitreal delivery of the Caspase-1 inhibitor Z-WEHD-FMK, blocked IL-18 maturation and Alu RNA induced RPE degeneration in wild type mice. Similarly, *Caspase-1*^–/–^ mice treated with Alu RNA did not exhibit RPE degeneration. In human RPE cells transfected with fluorescent tagged PYCARD (GFP-PYCARD), Alu RNA treatment induced NLRP3 inflammasome activation. Alu RNA didn’t induce RPE degeneration in either *Nlrp3*^–/–^ or *Pycard*^–/–^ mice, demonstrating the critical importance of the inflammasome in Alu RNA cytotoxicity. Pyroptosis can also proceed independent of IL-18. However, they found that IL-18 induced RPE degeneration in *Caspase-1*^–/–^ mice which couldn’t be rescued by a pyroptosis lysis inhibitor glycine. Therefore, they claimed that Alu RNA induced RPE degeneration does not occur via pyroptosis. In a later paper (Kim et al., 2014), they reported an increased total Caspase-8 protein level in the RPE of human eyes with GA compared with healthy, age-matched eyes. Since Caspase-8 can function either upstream or downstream of inflammasome activation, so they tested whether Caspase-8 is required for AluRNA induced RPE cell death and cytokine production. They observed Caspase-8 activation in primary human RPE cells treated with Alu RNA. RPE specific knock out of Dicer1 as well as Alu RNA subretinal injected wild type mice exhibited significantly greater Caspase-8 activation in RPE/choroid tissues while blockage of Caspase-8 protects RPE from Alu RNA toxicity. They then found that IL-18 induced RPE degeneration can be inhibited by knockout of Caspase-8. Also, subretinal injection of Alu RNA lead to elevated Fas ligand (FasL) and Fas receptor expression, which are required for the cleavage of pro-caspase 8 in RPE/choroid tissues; while *Fas* or *FasL* knock out inhibited Alu RNA induced RPE degeneration. Caspase-8 inhibitor failed to reduce Caspase-1 activation in Alu RNA treated human RPE cells which indicated that Caspase-8 acted downstream of Caspase-1. Previously, they reported Alu RNA induced activation of Caspase-3 in human RPE cells (Kaneko et al., 2011), a critical executioner in apoptotic cell death. In this paper, they showed Caspase-3 activation was induced by AluRNA in the RPE of wild type mice which could be inhibited by a Caspase-8 inhibitor. Taken together, they claimed that Caspase-8 functions between Caspase-1 and Caspase-3 in Alu RNA-induced RPE cell death process. In addition, they found that Nec-1, a necroptosis inhibitor, did not protect against Alu RNA-induced RPE degeneration. Overall, they suggest that Alu RNA/IL-18 induced Caspase-8 mediated RPE apoptosis in GA through Fas and FasL signaling in a non-cell autonomous manner. Yamada et al. reported that in human RPE cells, transfected with 2.5 nM Alu RNA for 96 h showed increasing expression of P16^INK4a^ and SA-β-Gal positivity (senescence markers) as well as expression of IL-18 and IL-1β (Yamada et al., 2020). However, more studies related to Alu RNA induced RPE cell senescence still need to be done to elucidate the mechanisms. The probability of pyroptosis in Alu RNA induced RPE death could be further examined besides the pyroptosis lysis inhibitor experiment. In addition, how Alu RNA leads to inflammasome activation could be further studied.

Some other RNA species, such as double strand RNA (dsRNA) analog poly(I : C), has been shown to induce necroptosis in RPE cells. RIPK3-dependent release of HMGB1 to the vitreous and TNF-α and IL-6 production were observed. In *Rip3*^–/–^ mice, both necrosis and inflammation were prevented. In RIPK3-deficient RPE cells, poly(I : C)-induced necrosis was inhibited which subsequently suppressed HMGB1 release and TNF-α and IL-6 induction. Cleavage of caspase-3 was observed in mice retina 2 days after poly(I : C) injection. However, Z-VAD did not show any additional significant protective effect on RPE or PRs in WT or *Rip3*^–/–^ mice after poly(I : C) injection. Therefore, necroptosis is more crucial in dsRNA-induced RPE degeneration (Murakami et al., 2014).

### Amyloid Beta (Aβ)

Amyloid beta peptide (Aβ) is a 37 to 49 amino acid residue peptide cleaved from amyloid precursor protein (APP) (Nunan and Small, 2000). The C-terminal fragment of APP (C99) is firstly generated by β-secretase cleavage, then γ-secretase cut C99 at multiple sites to produce fragments with different lengths that are further cleaved to the final Aβ forms. 40-amino-acid (Aβ1-40) and the 42-amino-acid (Aβ1-42) are two of the most abundant Aβ forms (Takami et al., 2009; Olsson et al., 2014). Aβ1-42 has a comparatively higher propensity to form prefibrillar aggregates and has been reported to be more toxic than Aβ1-40 (Dahlgren et al., 2002). Aβ is the main component of Amyloid plagues which are most commonly found in the neocortex in the brain of Alzheimer’s disease patients (Chen et al., 2017). Aβ1-42 is believed to induce oxidative stress during Alzheimer’s disease pathogenesis (Butterfield et al., 2013). Elevated Aβ levels have also been found in aged retina, and Aβ is also believed to play an role in the progression of AMD (Ohno-Matsui, 2011). Ye et al. (2018) detected apoptosis in RPE cells treated with 60 μM Aβ25–35 (a highly toxic Aβ peptides), shown as a significant increase in PI-negative and Annexin V-positive cells. The level of activated Caspase-3 protein, measured by western blotting, was markedly increased in cells treated with 60 μM Aβ25–35 for 36 h. Liu et al. (2014) demonstrated that Aβ1−40 stimulates chronic inflammation via NF-κB activation and plays an role in AMD pathology. They also found that Aβ1−40 induces inflammasome activation which in turn upregulates IL-6, TNF-α, IL-1β, IL-18, Caspase-1, and NLRP3 in RPE, choroid and neuroretina (Liu et al., 2013). Masuda et al. found Aβ increases the level of pigment epithelium-derived factor (PEDF) at a low concentration and thus inhibits the apoptosis of RPE cells. At a high concentration, Aβ induces Caspase-9 cascade in RPE cells and in turn leads to cell death. It also enhances VEGF-A transcription in RPE cells which may lead to the occurrence of CNV (Masuda et al., 2019). Gao et al. (2018) found that Aβ induces inflammasome activation and activates both pyroptosis and apoptosis RPE cells. Caspase-1 immunoreactivity was enhanced by 77% in the RPE layer of Aβ-injected rat eyes. IL-18 was elevated in the vitreous, showed a more than six folds higher immunoreactivity and a 58% increased band intensity in protein lysates in the RPE layer of Aβ-injected eyes, compared to the control eyes. RPE area was significantly increased in Aβ injected eyes, presumably due to swelling of the RPE cells. The cleaved N-terminal fragment of GSDMD (N-GSDMD) was increased while the uncleaved full-length GSDMD was decreased in the RPE/choroid tissue of Aβ-injected eyes. A higher immunoreactivity level of active Caspase-3 was shown in PR inner segments and RPE of the Aβ-injected eyes. X-chromosome-linked inhibitor of apoptosis (XIAP), a classic anti-apoptosis factor, was downregulated at both mRNA and protein levels in Aβ-injected eyes. Liu et al. (2015) showed increased p16^INK4a^ expression in Aβ1−42 subretinal injected mice RPE on day 7 post-injection which indicated the involvement of senescence. Based on these studies, apoptosis, pyroptosis and senescence could be all related to Aβ induced RPE degeneration. More experiments are needed to see the upstream and downstream of the Aβ induced metabolic changes and could give a hint about which cell death pathway is prominent. In a clinical trial, intravenous amyloid β inhibition with GSK933776 did not slow the rate of GA enlargement compared with placebo, and no meaningful differences relative to placebo were observed in visual function testing over 18 months (Rosenfeld et al., 2018). The potential of Aβ as therapeutic target for AMD should be further clarified.

### Sodium Iodate (NaIO_3_)

Sodium iodate injection-induced retinal degeneration displays features similar to AMD and has been used as a RPE dystrophy and GA model (Tang et al., 2013). NaIO_3_ induces ROS production and RPE damage and cell death. The affected RPE cells could cause secondary effects on PRs and the choriocapillaris (Noell, 1953; Korte et al., 1984). Additionally, NaIO_3_ could lead to the inhibition of enzyme activity in RPE cells and the disruption of the blood-retina barrier (Ashburn et al., 1980; Baich and Ziegler, 1992; Konda et al., 1994). In Mao et al. (2018) study, Annexin-V positive ARPE-19 cells increased from 1.7 to 8.8% during 48 h treatment with 1 mg/ml NaIO_3_. Increased expression and activity of Caspase-3 and -7 (shown by Western blotting) was also induced in NaIO_3_ treated ARPE-19 cells. They also showed upregulated expression of NLRP3, Caspase-1 and IL-1β, key molecules in pyroptosis, in NaIO_3_ treated ARPE-19 cells. However, co-culture with mesenchymal stem cells could suppress these effects. Moriguchi et al. (2018) using TUNEL staining showed that NaIO_3_ initially damage the RPE cells and then the neighboring PRs, which is consistent with the results of the Chen’s study (Chen et al., 2014). However, TUNEL assay identifies DNA break, not just apoptotic DNA ladder formation. Our lab has reported that 10 mM NaIO_3_ treatment could induce necrosome formation in ARPE-19 cells by transfecting the cells with a RIPK3-GFP-expressing plasmid. Under normal condition, RIPK3 was evenly distributed in the cytoplasm. With 2 h of 10 mM NaIO_3_ treatment, RIPK3 formed punctuates in the periphery region of cells which indicates necrosome formation. We also observed the release of HMGB1 to the cytoplasm and fragmented and clustered mitochondrial network within 4 h of NaIO_3_ treatment. Nec-1, Nec-5 and GSK’872 inhibited ARPE-19 cell death induced by NaIO_3_, while Nec-7 had no protective effect. Moreover, the pan-caspase inhibitor Z-VAD could not protect ARPE-19 cells from NaIO_3_ treatment. We didn’t see formation of inflammasomes in NaIO_3_-treated RPE cells. In addition, Caspase-1 inhibitor Ac-YVAD could not rescue RPE cells from NaIO_3_ treatment. Retro-orbital injection of NaIO_3_ at low dose (20 mg/kg) was also performed to mouse retina to test RPE cell death *in vivo*. RPE appeared swollen 72 h after the injection. Retro-orbital PI injection followed by retinal flat mount was then used to detect RPE necrosis in this model. PI-positive RPE cells began to appear at 24 h after NaIO_3_ injection. TUNEL-positive RPE cells started to show up at 24 h, increased at 48 h, then decreased at 72 h. Active Caspase-3 was detected only in PR layer but not in RPE cells by staining. RIPK3 aggregation was observed at 24 and 48 h after retro-orbital injection with NaIO_3_ in a transgenic mouse line expressing human RIPK3 in RPE cells. Also, Nec-1 inhibits RPE cell death *in vivo*. Based on our results, we propose that NaIO_3_ could induce RIPK1- and RIPK3-dependent necroptosis in RPE cells both *in vitro* and *in vivo* (Hanus et al., 2016). Ma et al. (2020) found that inhibition of thyroid hormone signaling protects RPE from NaIO_3_ induced necroptosis *in vivo*. Although NaIO3-induced RPE cell death cannot be completely interpreted as the mechanism of GA, the mechanism of NaIO3-induced RPE cell death still need to be clarified which could provide clues to understand how RPE could die and how RPE-damage mediates PRs and choriocapillaris damage in the context of GA.

## Conclusion Marks and Future Directions

Retinal pigment epithelial cells are critical for metabolism and homeostasis of retina. However, they are vulnerable to oxidative stress and other relevant stresses due to high metabolism, high exposure to light, oxidized POS and PUFAs. Together with aging, this could lead to RPE dysfunction, degeneration and AMD pathogenesis. Although apoptosis was initially suggested as the major mechanism of RPE cell death, RPE senescence and different modes of cell death has been recently studied in RPE cells under different AMD-relevant stress conditions. Different modes of cell death have been reported in RPE cells in response to H_2_O_2_, 4-HNE, NaIO_3_, A2E, Alu RNA, and Aβ, depending on the experimental conditions. Although apoptosis has been reported in RPE cells by all the stressors listed above, necrosis was also reported from recent studies, making the problem murky. For example, H_2_O_2_, NaIO_3_, and dsRNA could induce necroptosis, 4-HNE could induce ferroptosis, while Alu RNA, A2E, and Amyloid-β could induce pyroptosis. Moreover, all these stressors could induce RPE senescence. The actual fate of RPE cells in response to stresses likely depends on the type, dosage and duration of the stressors. In order to develop targeted therapy for AMD, it is important to identify the types of RPE cell death in response to stresses, since the implications from different cell deaths are different. For example, necrosis is inflammatory, but apoptosis is not. Further experiments are needed to ascertain the RPE cell fate and mechanism of RPE senescence and death *in vivo* and in AMD patients. Rounding, dissociation and sloughing of RPE cells have been observed in human atrophic AMD samples (Sarks et al., 1988; Curcio et al., 2017). The nature of RPE cell degeneration and death in AMD needs further study, although TUNEL positive “apoptotic” RPE cells were observed at the edge of atrophic areas in AMD (Dunaief et al., 2002). TUNEL assay has been used to identify apoptosis but it only detects DNA break that could happen in other types of cell death. A battery of molecular markers is needed to unequivocally determine the mechanism of RPE cell senescence and death in AMD. Also, oxidative stress has an interrelated relationship with inflammation. A bunch of inflammatory factors and cytokines could be activated in the context of oxidative stress which then leads to severer oxidants produce and subsequent cell death or senescence (Chatterjee, 2016). Thus, in future studies, it is also worthful to clarify the relationship between different stresses/cell death pathways and inflammation. Moreover, some of the *in vitro* and *in vivo* studies could be revisited with the more complete toolsets to clarify different modes of cell death. Regarding therapeutic development, caution should be taken whether alternative cell death pathway could be triggered if one type of cell death is inhibited. Cell-type specific mechanism of cell death should also be considered when performing mechanistic and therapeutic studies. For example, post-mitotic cells, like RPE cells that have limited regeneration potential, could be more resistant to apoptosis *in vivo* (Annis et al., 2016).

## Author Contributions

Both authors contributed in writing the review.

## Conflict of Interest

The authors declare that the research was conducted in the absence of any commercial or financial relationships that could be construed as a potential conflict of interest.

## References

[B1] Age-Related Eye Disease Study 2 Research (2013). Lutein + zeaxanthin and omega-3 fatty acids for age-related macular degeneration: the Age-Related Eye Disease Study 2 (AREDS2) randomized clinical trial. *JAMA* 309 2005–2015. 10.1001/jama.2013.4997 23644932

[B2] Age-Related Eye Disease Study Research (2001). A randomized, placebo-controlled, clinical trial of high-dose supplementation with vitamins C and E, beta carotene, and zinc for age-related macular degeneration and vision loss: AREDS report no. 8. *Arch. Ophthalmol.* 119 1417–1436. 10.1001/archopht.119.10.1417 11594942 PMC1462955

[B3] Alaimo A. Liñares G. G. Bujjamer J. M. Gorojod R. M. Alcon S. P. Martínez J. H. (2019). Toxicity of blue led light and A2E is associated to mitochondrial dynamics impairment in ARPE-19 cells: implications for age-related macular degeneration. *Arch. Toxicol.* 93 1401–1415. 10.1007/s00204-019-02409-6 30778631

[B4] Alge C. S. Priglinger S. G. Neubauer A. S. Kampik A. Zillig M. Bloemendal H. (2002). Retinal pigment epithelium is protected against apoptosis by alphaB-crystallin. *Invest. Ophthalmol. Vis. Sci.* 43 3575–3582. 12407170

[B5] Anderson O. A. Finkelstein A. Shima D. T. (2013). A2E induces IL-1ss production in retinal pigment epithelial cells via the NLRP3 inflammasome. *PLoS One* 8:e67263. 10.1371/journal.pone.0067263 23840644 PMC3696103

[B6] Annis R. P. Swahari V. Nakamura A. Xie A. X. Hammond S. M. Deshmukh M. (2016). Mature neurons dynamically restrict apoptosis via redundant premitochondrial brakes. *FEBS J.* 283 4569–4582. 10.1111/febs.13944 27797453 PMC5438853

[B7] Arthur J. R. (2000). The glutathione peroxidases. *Cell Mol. Life. Sci.* 57 1825–1835. 10.1007/pl00000664 11215509 PMC11147127

[B8] Ashburn F. S. Jr. Pilkerton A. R. Rao N. A. Marak G. E. (1980). The effects of iodate and iodoacetate on the retinal adhesion. *Invest. Ophthalmol. Vis. Sci.* 19 1427–1432. 7440100

[B9] Ayala A. Munoz M. F. Arguelles S. (2014). Lipid peroxidation: production, metabolism, and signaling mechanisms of malondialdehyde and 4-hydroxy-2-nonenal. *Oxid. Med. Cell. Longev.* 2014:360438. 10.1155/2014/360438 24999379 PMC4066722

[B10] Baas D. C. Despriet D. D. Gorgels T. G. Bergeron-Sawitzke J. Uitterlinden A. G. Hofman A. (2010). The ERCC6 gene and age-related macular degeneration. *PLoS One* 5:e13786. 10.1371/journal.pone.0013786 21072178 PMC2967476

[B11] Baich A. Ziegler M. (1992). The effect of sodium iodate and melanin on the formation of glyoxylate. *Pigment. Cell Res.* 5 394–395. 10.1111/j.1600-0749.1992.tb00568.x 1492073

[B12] Barak A. Morse L. S. Goldkorn T. (2001). Ceramide: a potential mediator of apoptosis in human retinal pigment epithelial cells. *Invest. Ophthalmol. Vis. Sci.* 42 247–254. 11133876

[B13] Beatty S. Koh H. Phil M. Henson D. Boulton M. (2000). The role of oxidative stress in the pathogenesis of age-related macular degeneration. *Surv. Ophthalmol.* 45 115–134. 10.1016/s0039-6257(00)00140-5 11033038

[B14] Beckman K. B. Ames B. N. (1998). The free radical theory of aging matures. *Physiol. Rev.* 78 547–581. 10.1152/physrev.1998.78.2.547 9562038

[B15] Bedard K. Krause K. H. (2007). The NOX family of ROS-generating NADPH oxidases: physiology and pathophysiology. *Physiol. Rev.* 87 245–313. 10.1152/physrev.00044.2005 17237347

[B16] Bergsbaken T. Fink S. L. Cookson B. T. (2009). Pyroptosis: host cell death and inflammation. *Nat. Rev. Microbiol.* 7 99–109. 10.1038/nrmicro2070 19148178 PMC2910423

[B17] Bernstein E. Caudy A. A. Hammond S. M. Hannon G. J. (2001). Role for a bidentate ribonuclease in the initiation step of RNA interference. *Nature* 409 363–366. 10.1038/35053110 11201747

[B18] Bernstein P. S. Law W. C. Rando R. R. (1987). Isomerization of all-trans-retinoids to 11-cis-retinoids in vitro. *Proc. Natl. Acad. Sci. U.S.A.* 84 1849–1853. 10.1073/pnas.84.7.1849 3494246 PMC304538

[B19] Bersuker K. Hendricks J. M. Li Z. Magtanong L. Ford B. Tang P. H. (2019). The CoQ oxidoreductase FSP1 acts parallel to GPX4 to inhibit ferroptosis. *Nature* 575 688–692. 10.1038/s41586-019-1705-2 31634900 PMC6883167

[B20] Birben E. Sahiner U. M. Sackesen C. Erzurum S. Kalayci O. (2012). Oxidative stress and antioxidant defense. *World Allergy Organ J.* 5 9–19. 10.1097/WOX.0b013e3182439613 23268465 PMC3488923

[B21] Bohr V. Anson R. M. Mazur S. Dianov G. (1998). Oxidative DNA damage processing and changes with aging. *Toxicol. Lett.* 102-103 47–52. 10.1016/s0378-4274(98)00280-x 10022231

[B22] Bohr V. A. Ottersen O. P. Tonjum T. (2007). Genome instability and DNA repair in brain, ageing and neurological disease. *Neuroscience* 145 1183–1186. 10.1016/j.neuroscience.2007.03.015 17400394

[B23] Bok D. (1985). Retinal photoreceptor-pigment epithelium interactions. Friedenwald lecture. *Invest. Ophthalmol. Vis. Sci.* 26 1659–1694. 2933359

[B24] Bonilha V. L. (2008). Age and disease-related structural changes in the retinal pigment epithelium. *Clin. Ophthalmol.* 2 413–424. 10.2147/opth.s2151 19668732 PMC2693982

[B25] Brand M. D. (2016). Mitochondrial generation of superoxide and hydrogen peroxide as the source of mitochondrial redox signaling. *Free Radic. Biol. Med.* 100 14–31. 10.1016/j.freeradbiomed.2016.04.001 27085844

[B26] Brentnall M. Rodriguez-Menocal L. De Guevara R. L. Cepero E. Boise L. H. (2013). Caspase-9, caspase-3 and caspase-7 have distinct roles during intrinsic apoptosis. *BMC Cell. Biol.* 14:32. 10.1186/1471-2121-14-32 23834359 PMC3710246

[B27] Bressler N. M. (2002). Early detection and treatment of neovascular age-related macular degeneration. *J. Am. Board. Fam. Pract.* 15 142–152. 12002198

[B28] Brigelius-Flohe R. Maiorino M. (2013). Glutathione peroxidases. *Biochim. Biophys. Acta* 1830 3289–3303. 10.1016/j.bbagen.2012.11.020 23201771

[B29] Brion M. Sanchez-Salorio M. Corton M. de la Fuente M. Pazos B. Othman M. (2011). Genetic association study of age-related macular degeneration in the Spanish population. *Acta Ophthalmol.* 89 e12–e22. 10.1111/j.1755-3768.2010.02040.x 21106043 PMC5745031

[B30] Burke J. M. (2008). Epithelial phenotype and the RPE: is the answer blowing in the Wnt? *Prog. Retin. Eye Res.* 27 579–595. 10.1016/j.preteyeres.2008.08.002 18775790 PMC2584165

[B31] Butterfield D. A. Swomley A. M. Sultana R. (2013). Amyloid beta-peptide (1-42)-induced oxidative stress in Alzheimer disease: importance in disease pathogenesis and progression. *Antioxid. Redox. Signal.* 19 823–835. 10.1089/ars.2012.5027 23249141 PMC3749710

[B32] Canli O. Alankus Y. B. Grootjans S. Vegi N. Hultner L. Hoppe P. S. (2016). Glutathione peroxidase 4 prevents necroptosis in mouse erythroid precursors. *Blood* 127 139–148. 10.1182/blood-2015-06-654194 26463424 PMC4705604

[B33] Cerbone A. Toaldo C. Laurora S. Briatore F. Pizzimenti S. Dianzani M. U. (2007). 4-Hydroxynonenal and PPARgamma ligands affect proliferation, differentiation, and apoptosis in colon cancer cells. *Free Radic. Biol. Med.* 42 1661–1670. 10.1016/j.freeradbiomed.2007.02.009 17462534

[B34] Chatterjee S. (2016). “Oxidative stress, inflammation, and disease,” in *Oxidative Stress and Biomaterials* (Academic Press), 35–58. 10.1016/B978-0-12-803269-5.00002-4

[B35] Chen C. Cano M. Wang J. J. Li J. Huang C. Yu Q. (2014). Role of unfolded protein response dysregulation in oxidative injury of retinal pigment epithelial cells. *Antioxid. Redox. Signal.* 20 2091–2106. 10.1089/ars.2013.5240 24053669 PMC3995121

[B36] Chen G. F. Xu T. H. Yan Y. Zhou Y. R. Jiang Y. Melcher K. (2017). Amyloid beta: structure, biology and structure-based therapeutic development. *Acta Pharmacol. Sin.* 38 1205–1235. 10.1038/aps.2017.28 28713158 PMC5589967

[B37] Chen Q. M. Liu J. Merrett J. B. (2000). Apoptosis or senescence-like growth arrest: influence of cell-cycle position, p53, p21 and bax in H2O2 response of normal human fibroblasts. *Biochem. J.* 347 543–551. 10.1042/0264-6021:3470543 10749685 PMC1220988

[B38] Childs B. G. Durik M. Baker D. J. van Deursen J. M. (2015). Cellular senescence in aging and age-related disease: from mechanisms to therapy. *Nat Med.* 21 1424–1435. 10.1038/nm.4000 26646499 PMC4748967

[B39] Cohen G. M. (1997). Caspases: the executioners of apoptosis. *Biochem. J.* 326 (Pt 1), 1–16. 10.1042/bj3260001 9337844 PMC1218630

[B40] Collado M. Serrano M. (2010). Senescence in tumours: evidence from mice and humans. *Nat. Rev. Cancer* 10 51–57. 10.1038/nrc2772 20029423 PMC3672965

[B41] Cordaux R. Batzer M. A. (2009). The impact of retrotransposons on human genome evolution. *Nat. Rev. Genet.* 10 691–703. 10.1038/nrg2640 19763152 PMC2884099

[B42] Crabb J. W. Miyagi M. Gu X. Shadrach K. West K. A. Sakaguchi H. (2002). Drusen proteome analysis: an approach to the etiology of age-related macular degeneration. *Proc. Natl. Acad. Sci. U.S.A.* 99 14682–14687. 10.1073/pnas.222551899 12391305 PMC137479

[B43] Curcio C. A. Zanzottera E. C. Ach T. Balaratnasingam C. Freund K. B. (2017). Activated retinal pigment epithelium, an optical coherence tomography biomarker for progression in age-related macular degeneration. *Invest. Ophthalmol. Vis. Sci.* 58 BIO211–BIO226. 10.1167/iovs.17-21872 28785769 PMC5557213

[B44] Dahlgren K. N. Manelli A. M. Stine W. B. Jr. Baker L. K. Krafft G. A. LaDu M. J. (2002). Oligomeric and fibrillar species of amyloid-beta peptides differentially affect neuronal viability. *J. Biol. Chem.* 277 32046–32053. 10.1074/jbc.M201750200 12058030

[B45] Datta S. Cano M. Ebrahimi K. Wang L. Handa J. T. (2017). The impact of oxidative stress and inflammation on RPE degeneration in non-neovascular AMD. *Prog. Retin. Eye Res.* 60 201–218. 10.1016/j.preteyeres.2017.03.002 28336424 PMC5600827

[B46] Davalli P. Mitic T. Caporali A. Lauriola A. D’Arca D. (2016). ROS, cell senescence, and novel molecular mechanisms in aging and age-related diseases. *Oxid. Med. Cell. Longev.* 2016:3565127. 10.1155/2016/3565127 27247702 PMC4877482

[B47] de Jong P. T. (2006). Age-related macular degeneration. *N. Engl. J. Med.* 355 1474–1485. 10.1056/NEJMra062326 17021323

[B48] Dimri G. P. Lee X. Basile G. Acosta M. Scott G. Roskelley C. (1995). A biomarker that identifies senescent human cells in culture and in aging skin in vivo. *Proc. Natl. Acad. Sci. U.S.A.* 92 9363–9367. 10.1073/pnas.92.20.9363 7568133 PMC40985

[B49] Ding J. Wang K. Liu W. She Y. Sun Q. Shi J. (2016). Pore-forming activity and structural autoinhibition of the gasdermin family. *Nature* 535 111–116. 10.1038/nature18590 27281216

[B50] Dixon S. J. Lemberg K. M. Lamprecht M. R. Skouta R. Zaitsev E. M. Gleason C. E. (2012). Ferroptosis: an iron-dependent form of nonapoptotic cell death. *Cell* 149 1060–1072. 10.1016/j.cell.2012.03.042 22632970 PMC3367386

[B51] Doll S. Freitas F. P. Shah R. Aldrovandi M. da Silva M. C. Ingold I. (2019). FSP1 is a glutathione-independent ferroptosis suppressor. *Nature* 575 693–698. 10.1038/s41586-019-1707-0 31634899

[B52] Dondelinger Y. Declercq W. Montessuit S. Roelandt R. Goncalves A. Bruggeman I. (2014). MLKL compromises plasma membrane integrity by binding to phosphatidylinositol phosphates. *Cell Rep.* 7 971–981. 10.1016/j.celrep.2014.04.026 24813885

[B53] Droge W. (2002). Free radicals in the physiological control of cell function. *Physiol. Rev.* 82 47–95. 10.1152/physrev.00018.2001 11773609

[B54] Du J. Yanagida A. Knight K. Engel A. L. Vo A. H. Jankowski C. (2016). Reductive carboxylation is a major metabolic pathway in the retinal pigment epithelium. *Proc. Natl. Acad. Sci. U.S.A.* 113 14710–14715. 10.1073/pnas.1604572113 27911769 PMC5187684

[B55] Dunaief J. L. Dentchev T. Ying G. S. Milam A. H. (2002). The role of apoptosis in age-related macular degeneration. *Arch. Ophthalmol.* 120 1435–1442. 10.1001/archopht.120.11.1435 12427055

[B56] Edwards A. O. Ritter R. III Abel K. J. Manning A. Panhuysen C. Farrer L. A. (2005). Complement factor H polymorphism and age-related macular degeneration. *Science* 308 421–424. 10.1126/science.1110189 15761121

[B57] Eguchi Y. Shimizu S. Tsujimoto Y. (1997). Intracellular ATP levels determine cell death fate by apoptosis or necrosis. *Cancer Res.* 57 1835–1840. 9157970

[B58] Elmore S. (2007). Apoptosis: a review of programmed cell death. *Toxicol Pathol.* 35 495–516. 10.1080/01926230701320337 17562483 PMC2117903

[B59] Esterbauer H. Schaur R. J. Zollner H. (1991). Chemistry and biochemistry of 4-hydroxynonenal, malonaldehyde and related aldehydes. *Free Radic. Biol. Med.* 11 81–128. 10.1016/0891-5849(91)90192-6 1937131

[B60] Ethen C. M. Reilly C. Feng X. Olsen T. W. Ferrington D. A. (2007). Age-related macular degeneration and retinal protein modification by 4-hydroxy-2-nonenal. *Invest. Ophthalmol. Vis. Sci.* 48 3469–3479. 10.1167/iovs.06-1058 17652714

[B61] Fang X. Wang H. Han D. Xie E. Yang X. Wei J. (2019). Ferroptosis as a target for protection against cardiomyopathy. *Proc. Natl. Acad. Sci. U.S.A.* 116 2672–2680. 10.1073/pnas.1821022116 30692261 PMC6377499

[B62] Ferrington D. A. Sinha D. Kaarniranta K. (2016). Defects in retinal pigment epithelial cell proteolysis and the pathology associated with age-related macular degeneration. *Prog. Retin. Eye Res.* 51 69–89. 10.1016/j.preteyeres.2015.09.002 26344735 PMC4769684

[B63] Fritsch M. Gunther S. D. Schwarzer R. Albert M. C. Schorn F. Werthenbach J. P. (2019). Caspase-8 is the molecular switch for apoptosis, necroptosis and pyroptosis. *Nature* 575 683–687. 10.1038/s41586-019-1770-6 31748744

[B64] Gao J. Cui J. Z. To E. Cao S. Matsubara J. A. (2018). Evidence for the activation of pyroptotic and apoptotic pathways in RPE cells associated with NLRP3 inflammasome in the rodent eye. *J. Neuroinflammation* 15:15. 10.1186/s12974-018-1062-3 29329580 PMC5766992

[B65] Gardner H. W. (1989). Oxygen radical chemistry of polyunsaturated fatty acids. *Free Radic. Biol. Med.* 7 65–86. 10.1016/0891-5849(89)90102-0 2666279

[B66] Giddabasappa A. Bauler M. Yepuru M. Chaum E. Dalton J. T. Eswaraka J. (2010). 17-beta estradiol protects ARPE-19 cells from oxidative stress through estrogen receptor-beta. *Invest. Ophthalmol. Vis. Sci.* 51 5278–5287. 10.1167/iovs.10-5316 20463317

[B67] Gil L. Siems W. Mazurek B. Gross J. Schroeder P. Voss P. (2006). Age-associated analysis of oxidative stress parameters in human plasma and erythrocytes. *Free Radic. Res.* 40 495–505. 10.1080/10715760600592962 16551576

[B68] Gong Y. N. Guy C. Olauson H. Becker J. U. Yang M. Fitzgerald P. (2017). ESCRT-III Acts Downstream of MLKL to regulate necroptotic cell death and its consequences. *Cell* 169 286.e16–300.e16. 10.1016/j.cell.2017.03.020 28388412 PMC5443414

[B69] Gray D. C. Mahrus S. Wells J. A. (2010). Activation of specific apoptotic caspases with an engineered small-molecule-activated protease. *Cell* 142 637–646. 10.1016/j.cell.2010.07.014 20723762 PMC3689538

[B70] Haines J. L. Hauser M. A. Schmidt S. Scott W. K. Olson L. M. Gallins P. (2005). Complement factor H variant increases the risk of age-related macular degeneration. *Science* 308 419–421. 10.1126/science.1110359 15761120

[B71] Halliwell B. Chirico S. (1993). Lipid peroxidation: its mechanism, measurement, and significance. *Am. J. Clin. Nutr.* 57 715S–724S. 10.1093/ajcn/57.5.715S 8475889

[B72] Halliwell B. Gutteridge J. M. (1986). Oxygen free radicals and iron in relation to biology and medicine: some problems and concepts. *Arch. Biochem. Biophys.* 246 501–514. 10.1016/0003-9861(86)90305-x 3010861

[B73] Hampel B. Malisan F. Niederegger H. Testi R. Jansen-Durr P. (2004). Differential regulation of apoptotic cell death in senescent human cells. *Exp. Gerontol.* 39 1713–1721. 10.1016/j.exger.2004.05.010 15582287

[B74] Hanus J. Anderson C. Sarraf D. Ma J. Wang S. (2016). Retinal pigment epithelial cell necroptosis in response to sodium iodate. *Cell Death Discov.* 2:16054. 10.1038/cddiscovery.2016.54 27551542 PMC4979458

[B75] Hanus J. Zhang H. Wang Z. Liu Q. Zhou Q. Wang S. (2013). Induction of necrotic cell death by oxidative stress in retinal pigment epithelial cells. *Cell Death Dis.* 4:e965. 10.1038/cddis.2013.478 24336085 PMC3877549

[B76] Hayflick L. (1965). The limited in vitro lifetime of human diploid cell strains. *Exp. Cell Res.* 37 614–636. 10.1016/0014-4827(65)90211-9 14315085

[B77] He W. T. Wan H. Hu L. Chen P. Wang X. Huang Z. (2015). Gasdermin D is an executor of pyroptosis and required for interleukin-1beta secretion. *Cell. Res.* 25 1285–1298. 10.1038/cr.2015.139 26611636 PMC4670995

[B78] Herbig U. Jobling W. A. Chen B. P. Chen D. J. Sedivy J. M. (2004). Telomere shortening triggers senescence of human cells through a pathway involving ATM, p53, and p21(CIP1), but not p16(INK4a). *Mol. Cell.* 14 501–513. 10.1016/s1097-2765(04)00256-4 15149599

[B79] Hitomi J. Katayama T. Eguchi Y. Kudo T. Taniguchi M. Koyama Y. (2004). Involvement of caspase-4 in endoplasmic reticulum stress-induced apoptosis and Abeta-induced cell death. *J. Cell Biol.* 165 347–356. 10.1083/jcb.200310015 15123740 PMC2172196

[B80] Hollyfield J. G. Bonilha V. L. Rayborn M. E. Yang X. Shadrach K. G. Lu L. (2008). Oxidative damage-induced inflammation initiates age-related macular degeneration. *Nat. Med.* 14 194–198. 10.1038/nm1709 18223656 PMC2748836

[B81] Holtkamp G. M. Kijlstra A. Peek R. de Vos A. F. (2001). Retinal pigment epithelium-immune system interactions: cytokine production and cytokine-induced changes. *Prog. Retin. Eye Res.* 20 29–48. 10.1016/s1350-9462(00)00017-3 11070367

[B82] Hwang Y. E. Baek Y. M. Baek A. Kim D. E. (2019). Oxidative stress causes Alu RNA accumulation via PIWIL4 sequestration into stress granules. *BMB Rep.* 52 196–201. 10.5483/bmbrep.2019.52.3.146 30103846 PMC6476485

[B83] Igney F. H. Krammer P. H. (2002). Death and anti-death: tumour resistance to apoptosis. *Nat. Rev. Cancer* 2 277–288. 10.1038/nrc776 12001989

[B84] Jager R. D. Mieler W. F. Miller J. W. (2008). Age-related macular degeneration. *N. Engl. J. Med.* 358 2606–2617. 10.1056/NEJMra0801537 18550876

[B85] Jarrett S. G. Boulton M. E. (2012). Consequences of oxidative stress in age-related macular degeneration. *Mol. Aspects Med.* 33 399–417. 10.1016/j.mam.2012.03.009 22510306 PMC3392472

[B86] Jonasson F. Arnarsson A. Eiriksdottir G. Harris T. B. Launer L. J. Meuer S. M. (2011). Prevalence of age-related macular degeneration in old persons: age, gene/environment susceptibility reykjavik Study. *Ophthalmology* 118 825–830. 10.1016/j.ophtha.2010.08.044 21126770 PMC3087833

[B87] Kaneko H. Dridi S. Tarallo V. Gelfand B. D. Fowler B. J. Cho W. G. (2011). DICER1 deficit induces Alu RNA toxicity in age-related macular degeneration. *Nature* 471 325–330. 10.1038/nature09830 21297615 PMC3077055

[B88] Kang T. B. Yang S. H. Toth B. Kovalenko A. Wallach D. (2013). Caspase-8 blocks kinase RIPK3-mediated activation of the NLRP3 inflammasome. *Immunity* 38 27–40. 10.1016/j.immuni.2012.09.015 23260196

[B89] Kayagaki N. Warming S. Lamkanfi M. Vande Walle L. Louie S. Dong J. (2011). Non-canonical inflammasome activation targets caspase-11. *Nature* 479 117–121. 10.1038/nature10558 22002608

[B90] Kerr J. F. Wyllie A. H. Currie A. R. (1972). Apoptosis: a basic biological phenomenon with wide-ranging implications in tissue kinetics. *Br. J. Cancer* 26 239–257. 10.1038/bjc.1972.33 4561027 PMC2008650

[B91] Khandhadia S. Lotery A. (2010). Oxidation and age-related macular degeneration: insights from molecular biology. *Expert Rev. Mol. Med.* 12:e34. 10.1017/S146239941000164X 20959033

[B92] Khosla S. Farr J. N. Tchkonia T. Kirkland J. L. (2020). The role of cellular senescence in ageing and endocrine disease. *Nat. Rev. Endocrinol.* 16 263–275. 10.1038/s41574-020-0335-y 32161396 PMC7227781

[B93] Kilinc A. Yalcin A. S. Yalcin D. Taga Y. Emerk K. (1988). Increased erythrocyte susceptibility to lipid peroxidation in human Parkinson’s disease. *Neurosci. Lett.* 87 307–310. 10.1016/0304-3940(88)90467-3 3380350

[B94] Kim M. H. Chung J. Yang J. W. Chung S. M. Kwag N. H. Yoo J. S. (2003). Hydrogen peroxide-induced cell death in a human retinal pigment epithelial cell line, ARPE-19. *Korean J. Ophthalmol.* 17 19–28. 10.3341/kjo.2003.17.1.19 12882504

[B95] Kim Y. Tarallo V. Kerur N. Yasuma T. Gelfand B. D. Bastos-Carvalho A. (2014). DICER1/Alu RNA dysmetabolism induces Caspase-8-mediated cell death in age-related macular degeneration. *Proc. Natl. Acad. Sci. U.S.A.* 111 16082–16087. 10.1073/pnas.1403814111 25349431 PMC4234570

[B96] Klein R. Klein B. E. Linton K. L. DeMets D. L. (1993). The Beaver Dam Eye Study: the relation of age-related maculopathy to smoking. *Am. J. Epidemiol.* 137 190–200. 10.1093/oxfordjournals.aje.a116659 8452123

[B97] Klein R. Klein B. E. Moss S. E. (1998). Relation of smoking to the incidence of age-related maculopathy. The beaver dam eye study. *Am. J. Epidemiol.* 147 103–110. 10.1093/oxfordjournals.aje.a009421 9456998

[B98] Klein R. J. Zeiss C. Chew E. Y. Tsai J. Y. Sackler R. S. Haynes C. (2005). Complement factor H polymorphism in age-related macular degeneration. *Science* 308 385–389. 10.1126/science.1109557 15761122 PMC1512523

[B99] Konda B. R. Pararajasegaram G. Wu G. S. Stanforth D. Rao N. A. (1994). Role of retinal pigment epithelium in the development of experimental autoimmune uveitis. *Invest. Ophthalmol. Vis. Sci.* 35 40–47. 8300362

[B100] Korte G. E. Reppucci V. Henkind P. (1984). RPE destruction causes choriocapillary atrophy. *Invest. Ophthalmol. Vis. Sci.* 25 1135–1145. 6480292

[B101] Kovacs S. B. Miao E. A. (2017). Gasdermins: effectors of pyroptosis. *Trends Cell Biol.* 27 673–684. 10.1016/j.tcb.2017.05.005 28619472 PMC5565696

[B102] Kuilman T. Michaloglou C. Mooi W. J. Peeper D. S. (2010). The essence of senescence. *Genes Dev.* 24 2463–2479. 10.1101/gad.1971610 21078816 PMC2975923

[B103] Laurora S. Tamagno E. Briatore F. Bardini P. Pizzimenti S. Toaldo C. (2005). 4-Hydroxynonenal modulation of p53 family gene expression in the SK-N-BE neuroblastoma cell line. *Free Radic. Biol. Med.* 38 215–225. 10.1016/j.freeradbiomed.2004.10.014 15607904

[B104] Lee B. Y. Han J. A. Im J. S. Morrone A. Johung K. Goodwin E. C. (2006). Senescence-associated beta-galactosidase is lysosomal beta-galactosidase. *Aging Cell* 5 187–195. 10.1111/j.1474-9726.2006.00199.x 16626397

[B105] Li G. Y. Fan B. Zheng Y. C. (2010). Calcium overload is a critical step in programmed necrosis of ARPE-19 cells induced by high-concentration H2O2. *Biomed. Environ. Sci.* 23 371–377. 10.1016/S0895-3988(10)60078-5 21112485

[B106] Li J. Cao F. Yin H. L. Huang Z. J. Lin Z. T. Mao N. (2020). Ferroptosis: past, present and future. *Cell Death Dis.* 11:88. 10.1038/s41419-020-2298-2 32015325 PMC6997353

[B107] Liu C. Cao L. Yang S. Xu L. Liu P. Wang F. (2015). Subretinal injection of amyloid-beta peptide accelerates RPE cell senescence and retinal degeneration. *Int. J. Mol. Med.* 35 169–176. 10.3892/ijmm.2014.1993 25385658

[B108] Liu R. T. Gao J. Cao S. Sandhu N. Cui J. Z. Chou C. L. (2013). Inflammatory mediators induced by amyloid-beta in the retina and RPE in vivo: implications for inflammasome activation in age-related macular degeneration. *Invest. Ophthalmol. Vis. Sci.* 54 2225–2237. 10.1167/iovs.12-10849 23462752 PMC3947398

[B109] Liu R. T. Wang A. To E. Gao J. Cao S. Cui J. Z. (2014). Vinpocetine inhibits amyloid-beta induced activation of NF-kappaB, NLRP3 inflammasome and cytokine production in retinal pigment epithelial cells. *Exp. Eye Res.* 127 49–58. 10.1016/j.exer.2014.07.003 25041941 PMC4461375

[B110] Liu X. Lieberman J. (2017). A mechanistic understanding of pyroptosis: the fiery death triggered by invasive infection. *Adv. Immunol.* 135 81–117. 10.1016/bs.ai.2017.02.002 28826530 PMC10245508

[B111] Liu X. Zhang Z. Ruan J. Pan Y. Magupalli V. G. Wu H. (2016). Inflammasome-activated gasdermin D causes pyroptosis by forming membrane pores. *Nature* 535 153–158. 10.1038/nature18629 27383986 PMC5539988

[B112] Luo X. Gu S. Zhang Y. Zhang J. (2018). Kinsenoside Ameliorates Oxidative Stress-Induced RPE Cell Apoptosis and Inhibits Angiogenesis via Erk/p38/NF-kappaB/VEGF Signaling. *Front. Pharmacol.* 9:240. 10.3389/fphar.2018.00240 29615910 PMC5870051

[B113] Ma H. Yang F. Ding X. Q. (2020). Inhibition of thyroid hormone signaling protects retinal pigment epithelium and photoreceptors from cell death in a mouse model of age-related macular degeneration. *Cell Death Dis.* 11:24. 10.1038/s41419-019-2216-7 31932580 PMC6957507

[B114] Mao X. Pan T. Shen H. Xi H. Yuan S. Liu Q. (2018). The rescue effect of mesenchymal stem cell on sodium iodate-induced retinal pigment epithelial cell death through deactivation of NF-kappaB-mediated NLRP3 inflammasome. *Biomed. Pharmacother.* 103 517–523. 10.1016/j.biopha.2018.04.038 29677537

[B115] Marazita M. C. Dugour A. Marquioni-Ramella M. D. Figueroa J. M. Suburo A. M. (2016). Oxidative stress-induced premature senescence dysregulates VEGF and CFH expression in retinal pigment epithelial cells: implications for age-related macular degeneration. *Redox Biol.* 7 78–87. 10.1016/j.redox.2015.11.011 26654980 PMC4683426

[B116] Martin-Sanchez D. Ruiz-Andres O. Poveda J. Carrasco S. Cannata-Ortiz P. Sanchez-Nino M. D. (2017). Ferroptosis, but not necroptosis, is important in nephrotoxic folic acid-induced AKI. *J. Am. Soc. Nephrol.* 28 218–229. 10.1681/ASN.2015121376 27352622 PMC5198282

[B117] Masuda N. Tsujinaka H. Hirai H. Yamashita M. Ueda T. Ogata N. (2019). Effects of concentration of amyloid beta (Abeta) on viability of cultured retinal pigment epithelial cells. *BMC Ophthalmol.* 19:70. 10.1186/s12886-019-1076-3 30849957 PMC6408759

[B118] McCord J. M. (2000). The evolution of free radicals and oxidative stress. *Am. J. Med.* 108 652–659. 10.1016/s0002-9343(00)00412-5 10856414

[B119] Mettu P. S. Wielgus A. R. Ong S. S. Cousins S. W. (2012). Retinal pigment epithelium response to oxidant injury in the pathogenesis of early age-related macular degeneration. *Mol. Aspects Med.* 33 376–398. 10.1016/j.mam.2012.04.006 22575354

[B120] Micklisch S. Lin Y. Jacob S. Karlstetter M. Dannhausen K. Dasari P. (2017). Age-related macular degeneration associated polymorphism rs10490924 in ARMS2 results in deficiency of a complement activator. *J. Neuroinflammation* 14:4. 10.1186/s12974-016-0776-3 28086806 PMC5234120

[B121] Moriguchi M. Nakamura S. Inoue Y. Nishinaka A. Nakamura M. Shimazawa M. (2018). Irreversible photoreceptors and RPE cells damage by intravenous sodium iodate in mice is related to macrophage accumulation. *Invest. Ophthalmol. Vis. Sci.* 59 3476–3487. 10.1167/iovs.17-23532 30025075

[B122] Muller T. Dewitz C. Schmitz J. Schroder A. S. Brasen J. H. Stockwell B. R. (2017). Necroptosis and ferroptosis are alternative cell death pathways that operate in acute kidney failure. *Cell Mol. Life Sci.* 74 3631–3645. 10.1007/s00018-017-2547-4 28551825 PMC5589788

[B123] Murakami Y. Matsumoto H. Roh M. Giani A. Kataoka K. Morizane Y. (2014). Programmed necrosis, not apoptosis, is a key mediator of cell loss and DAMP-mediated inflammation in dsRNA-induced retinal degeneration. *Cell Death Differ.* 21 270–277. 10.1038/cdd.2013.109 23954861 PMC3890945

[B124] Nair U. Bartsch H. Nair J. (2007). Lipid peroxidation-induced DNA damage in cancer-prone inflammatory diseases: a review of published adduct types and levels in humans. *Free Radic. Biol. Med.* 43 1109–1120. 10.1016/j.freeradbiomed.2007.07.012 17854706

[B125] Negre-Salvayre A. Coatrieux C. Ingueneau C. Salvayre R. (2008). Advanced lipid peroxidation end products in oxidative damage to proteins. Potential role in diseases and therapeutic prospects for the inhibitors. *Br. J. Pharmacol.* 153 6–20. 10.1038/sj.bjp.0707395 17643134 PMC2199390

[B126] Nelson G. Wordsworth J. Wang C. Jurk D. Lawless C. Martin-Ruiz C. (2012). A senescent cell bystander effect: senescence-induced senescence. *Aging Cell* 11 345–349. 10.1111/j.1474-9726.2012.00795.x 22321662 PMC3488292

[B127] Ngai L. Y. Stocks N. Sparrow J. M. Patel R. Rumley A. Lowe G. (2011). The prevalence and analysis of risk factors for age-related macular degeneration: 18-year follow-up data from the Speedwell eye study, United Kingdom. *Eye* 25 784–793. 10.1038/eye.2011.56 21436849 PMC3178119

[B128] Niki E. (2009). Lipid peroxidation: physiological levels and dual biological effects. *Free Radic. Biol. Med.* 47 469–484. 10.1016/j.freeradbiomed.2009.05.032 19500666

[B129] Noell W. K. (1953). Experimentally induced toxic effects on structure and function of visual cells and pigment epithelium. *Am. J. Ophthalmol.* 36 103–116. 10.1016/0002-9394(53)90159-7 13050703

[B130] Nunan J. Small D. H. (2000). Regulation of APP cleavage by alpha-, beta- and gamma-secretases. *FEBS Lett.* 483 6–10. 10.1016/s0014-5793(00)02076-7 11033346

[B131] Ogryzko V. V. Hirai T. H. Russanova V. R. Barbie D. A. Howard B. H. (1996). Human fibroblast commitment to a senescence-like state in response to histone deacetylase inhibitors is cell cycle dependent. *Mol. Cell. Biol.* 16 5210–5218. 10.1128/mcb.16.9.5210 8756678 PMC231521

[B132] Ohno-Matsui K. (2011). Parallel findings in age-related macular degeneration and Alzheimer’s disease. *Prog. Retin. Eye Res.* 30 217–238. 10.1016/j.preteyeres.2011.02.004 21440663

[B133] Olsson F. Schmidt S. Althoff V. Munter L. M. Jin S. Rosqvist S. (2014). Characterization of intermediate steps in amyloid beta (Abeta) production under near-native conditions. *J. Biol. Chem.* 289 1540–1550. 10.1074/jbc.M113.498246 24225948 PMC3894335

[B134] Pandey R. Mandal A. K. Jha V. Mukerji M. (2011). Heat shock factor binding in Alu repeats expands its involvement in stress through an antisense mechanism. *Genome Biol.* 12:R117. 10.1186/gb-2011-12-11-r117 22112862 PMC3334603

[B135] Panning B. Smiley J. R. (1994). Activation of RNA polymerase III transcription of human Alu elements by herpes simplex virus. *Virology* 202 408–417. 10.1006/viro.1994.1357 8009851

[B136] Pascolini D. Mariotti S. P. (2012). Global estimates of visual impairment: 2010. *Br. J. Ophthalmol.* 96 614–618. 10.1136/bjophthalmol-2011-300539 22133988

[B137] Phaniendra A. Jestadi D. B. Periyasamy L. (2015). Free radicals: properties, sources, targets, and their implication in various diseases. *Indian J. Clin. Biochem.* 30 11–26. 10.1007/s12291-014-0446-0 25646037 PMC4310837

[B138] Pizzino G. Irrera N. Cucinotta M. Pallio G. Mannino F. Arcoraci V. (2017). Oxidative stress: harms and benefits for human health. *Oxid. Med. Cell. Longev.* 2017:8416763. 10.1155/2017/8416763 28819546 PMC5551541

[B139] Rajendran P. Nandakumar N. Rengarajan T. Palaniswami R. Gnanadhas E. N. Lakshminarasaiah U. (2014). Antioxidants and human diseases. *Clin. Chim. Acta* 436 332–347. 10.1016/j.cca.2014.06.004 24933428

[B140] Richter C. Schweizer M. Cossarizza A. Franceschi C. (1996). Control of apoptosis by the cellular ATP level. *FEBS Lett.* 378 107–110. 10.1016/0014-5793(95)01431-4 8549813

[B141] Rodriguez D. A. Weinlich R. Brown S. Guy C. Fitzgerald P. Dillon C. P. (2016). Characterization of RIPK3-mediated phosphorylation of the activation loop of MLKL during necroptosis. *Cell Death Differ.* 23 76–88. 10.1038/cdd.2015.70 26024392 PMC4815980

[B142] Rosenfeld P. J. Berger B. Reichel E. Danis R. P. Gress A. Ye L. (2018). A randomized phase 2 study of an anti-amyloid beta monoclonal antibody in geographic atrophy secondary to age-related macular degeneration. *Ophthalmol. Retina* 2 1028–1040. 10.1016/j.oret.2018.03.001 31047490

[B143] Sarks J. P. Sarks S. H. Killingsworth M. C. (1988). Evolution of geographic atrophy of the retinal pigment epithelium. *Eye* 2 (Pt 5), 552–577. 10.1038/eye.1988.106 2476333

[B144] Schaur R. J. Siems W. Bresgen N. Eckl P. M. (2015). 4-Hydroxy-nonenal-a bioactive lipid peroxidation product. *Biomolecules* 5 2247–2337. 10.3390/biom5042247 26437435 PMC4693237

[B145] Schutt F. Bergmann M. Holz F. G. Kopitz J. (2003). Proteins modified by malondialdehyde, 4-hydroxynonenal, or advanced glycation end products in lipofuscin of human retinal pigment epithelium. *Invest. Ophthalmol. Vis. Sci.* 44 3663–3668. 10.1167/iovs.03-0172 12882821

[B146] Shaban H. Borras C. Vina J. Richter C. (2002). Phosphatidylglycerol potently protects human retinal pigment epithelial cells against apoptosis induced by A2E, a compound suspected to cause age-related macula degeneration. *Exp. Eye Res.* 75 99–108. 10.1006/exer.2001.1192 12123641

[B147] Shaban H. Gazzotti P. Richter C. (2001). Cytochrome c oxidase inhibition by N-retinyl-N-retinylidene ethanolamine, a compound suspected to cause age-related macula degeneration. *Arch. Biochem. Biophys.* 394 111–116. 10.1006/abbi.2001.2535 11566033

[B148] Sharma A. Sharma R. Chaudhary P. Vatsyayan R. Pearce V. Jeyabal P. V. (2008). 4-Hydroxynonenal induces p53-mediated apoptosis in retinal pigment epithelial cells. *Arch. Biochem. Biophys.* 480 85–94. 10.1016/j.abb.2008.09.016 18930016 PMC2664083

[B149] Shi J. Zhao Y. Wang Y. Gao W. Ding J. Li P. (2014). Inflammatory caspases are innate immune receptors for intracellular LPS. *Nature* 514 187–192. 10.1038/nature13683 25119034

[B150] Shoeb M. Ansari N. H. Srivastava S. K. Ramana K. V. (2014). 4-Hydroxynonenal in the pathogenesis and progression of human diseases. *Curr. Med. Chem.* 21 230–237. 10.2174/09298673113209990181 23848536 PMC3964795

[B151] Sies H. Chance B. (1970). The steady state level of catalase compound I in isolated hemoglobin-free perfused rat liver. *FEBS Lett.* 11 172–176. 10.1016/0014-5793(70)80521-x 11945479

[B152] Simon J. D. Hong L. Peles D. N. (2008). Insights into melanosomes and melanin from some interesting spatial and temporal properties. *J. Phys. Chem. B* 112 13201–13217. 10.1021/jp804248h 18817437

[B153] Skoumalova A. Hort J. (2012). Blood markers of oxidative stress in Alzheimer’s disease. *J. Cell. Mol. Med.* 16 2291–2300. 10.1111/j.1582-4934.2012.01585.x 22564475 PMC3823422

[B154] Sparrow J. R. Boulton M. (2005). RPE lipofuscin and its role in retinal pathobiology. *Exp. Eye Res.* 80 595–606. 10.1016/j.exer.2005.01.007 15862166

[B155] Sparrow J. R. Cai B. (2001). Blue light-induced apoptosis of A2E-containing RPE: involvement of caspase-3 and protection by Bcl-2. *Invest. Ophthalmol. Vis. Sci.* 42 1356–1362. 11328751

[B156] Sparrow J. R. Fishkin N. Zhou J. Cai B. Jang Y. P. Krane S. (2003a). A2E, a byproduct of the visual cycle. *Vis. Res.* 43 2983–2990. 10.1016/s0042-6989(03)00475-9 14611934

[B157] Sparrow J. R. Hicks D. Hamel C. P. (2010). The retinal pigment epithelium in health and disease. *Curr. Mol. Med.* 10 802–823. 10.2174/156652410793937813 21091424 PMC4120883

[B158] Sparrow J. R. Vollmer-Snarr H. R. Zhou J. Jang Y. P. Jockusch S. Itagaki Y. (2003b). A2E-epoxides damage DNA in retinal pigment epithelial cells. Vitamin E and other antioxidants inhibit A2E-epoxide formation. *J. Biol. Chem.* 278 18207–18213. 10.1074/jbc.M300457200 12646558

[B159] Spiteller P. Kern W. Reiner J. Spiteller G. (2001). Aldehydic lipid peroxidation products derived from linoleic acid. *Biochim. Biophys. Acta* 1531 188–208. 10.1016/s1388-1981(01)00100-7 11325611

[B160] Sreekumar P. G. Kannan R. Yaung J. Spee C. K. Ryan S. J. Hinton D. R. (2005). Protection from oxidative stress by methionine sulfoxide reductases in RPE cells. *Biochem. Biophys. Res. Commun.* 334 245–253. 10.1016/j.bbrc.2005.06.081 15993845

[B161] Stadtman E. R. Levine R. L. (2000). Protein oxidation. *Ann. N. Y. Acad. Sci.* 899 191–208. 10.1111/j.1749-6632.2000.tb06187.x 10863540

[B162] Sun L. Wang H. Wang Z. He S. Chen S. Liao D. (2012). Mixed lineage kinase domain-like protein mediates necrosis signaling downstream of RIP3 kinase. *Cell* 148 213–227. 10.1016/j.cell.2011.11.031 22265413

[B163] Suzuki M. Kamei M. Itabe H. Yoneda K. Bando H. Kume N. (2007). Oxidized phospholipids in the macula increase with age and in eyes with age-related macular degeneration. *Mol. Vis.* 13 772–778. 17563727 PMC2768762

[B164] Szabo C. Ischiropoulos H. Radi R. (2007). Peroxynitrite: biochemistry, pathophysiology and development of therapeutics. *Nat. Rev. Drug Discov.* 6 662–680. 10.1038/nrd2222 17667957

[B165] Takami M. Nagashima Y. Sano Y. Ishihara S. Morishima-Kawashima M. Funamoto S. (2009). gamma-Secretase: successive tripeptide and tetrapeptide release from the transmembrane domain of beta-carboxyl terminal fragment. *J. Neurosci.* 29 13042–13052. 10.1523/JNEUROSCI.2362-09.2009 19828817 PMC6665297

[B166] Tang P. H. Kono M. Koutalos Y. Ablonczy Z. Crouch R. K. (2013). New insights into retinoid metabolism and cycling within the retina. *Prog. Retin. Eye Res.* 32 48–63. 10.1016/j.preteyeres.2012.09.002 23063666 PMC3746031

[B167] Tarallo V. Hirano Y. Gelfand B. D. Dridi S. Kerur N. Kim Y. (2012). DICER1 loss and Alu RNA induce age-related macular degeneration via the NLRP3 inflammasome and MyD88. *Cell* 149 847–859. 10.1016/j.cell.2012.03.036 22541070 PMC3351582

[B168] Therond P. (2006). [Oxidative stress and damages to biomolecules (lipids, proteins, DNA)]. *Ann. Pharm. Fr.* 64 383–389. 10.1016/s0003-4509(06)75333-0 17119467

[B169] Tuo J. Bojanowski C. M. Chan C. C. (2004). Genetic factors of age-related macular degeneration. *Prog. Retin. Eye Res.* 23 229–249. 10.1016/j.preteyeres.2004.02.001 15094132 PMC1950336

[B170] Valko M. Leibfritz D. Moncol J. Cronin M. T. Mazur M. Telser J. (2007). Free radicals and antioxidants in normal physiological functions and human disease. *Int. J. Biochem. Cell Biol.* 39 44–84. 10.1016/j.biocel.2006.07.001 16978905

[B171] van Deursen J. M. (2014). The role of senescent cells in ageing. *Nature* 509 439–446. 10.1038/nature13193 24848057 PMC4214092

[B172] Wang J. Feng Y. Han P. Wang F. Luo X. Liang J. (2018). Photosensitization of A2E triggers telomere dysfunction and accelerates retinal pigment epithelium senescence. *Cell Death Dis.* 9:178. 10.1038/s41419-017-0200-7 29415988 PMC5833825

[B173] Wang W. Wang W. H. Azadzoi K. M. Dai P. Wang Q. Sun J. B. (2016). Alu RNA accumulation in hyperglycemia augments oxidative stress and impairs eNOS and SOD2 expression in endothelial cells. *Mol. Cell. Endocrinol.* 426 91–100. 10.1016/j.mce.2016.02.008 26891959

[B174] Wang Y. Y. Liu X. L. Zhao R. (2019). Induction of pyroptosis and its implications in cancer management. *Front. Oncol.* 9:971. 10.3389/fonc.2019.00971 31616642 PMC6775187

[B175] Weinlich R. Oberst A. Beere H. M. Green D. R. (2017). Necroptosis in development, inflammation and disease. *Nat. Rev. Mol. Cell Biol.* 18 127–136. 10.1038/nrm.2016.149 27999438

[B176] Weiter J. J. Delori F. C. Wing G. L. Fitch K. A. (1986). Retinal pigment epithelial lipofuscin and melanin and choroidal melanin in human eyes. *Invest. Ophthalmol. Vis. Sci.* 27 145–152. 3943941

[B177] Wielgus A. R. Collier R. J. Martin E. Lih F. B. Tomer K. B. Chignell C. F. (2010). Blue light induced A2E oxidation in rat eyes–experimental animal model of dry AMD. *Photochem. Photobiol. Sci.* 9 1505–1512. 10.1039/c0pp00133c 20922251 PMC12384401

[B178] Wimmers S. Karl M. O. Strauss O. (2007). Ion channels in the RPE. *Prog. Retin. Eye Res.* 26 263–301. 10.1016/j.preteyeres.2006.12.002 17258931

[B179] Winkler B. S. Boulton M. E. Gottsch J. D. Sternberg P. (1999). Oxidative damage and age-related macular degeneration. *Mol. Vis.* 5:32. PMC1773059 10562656

[B180] Wong W. L. Su X. Li X. Cheung C. M. Klein R. Cheng C. Y. (2014). Global prevalence of age-related macular degeneration and disease burden projection for 2020 and 2040: a systematic review and meta-analysis. *Lancet Glob. Health* 2 e106–e116. 10.1016/S2214-109X(13)70145-1 25104651

[B181] Xie X. Feng J. Kang Z. Zhang S. Zhang L. Zhang Y. (2017). Taxifolin protects RPE cells against oxidative stress-induced apoptosis. *Mol. Vis.* 23 520–528. 28761325 PMC5534490

[B182] Xu Y. J. Zheng L. Hu Y. W. Wang Q. (2018). Pyroptosis and its relationship to atherosclerosis. *Clin. Chim. Acta* 476 28–37. 10.1016/j.cca.2017.11.005 29129476

[B183] Yagoda N. von Rechenberg M. Zaganjor E. Bauer A. J. Yang W. S. Fridman D. J. (2007). RAS-RAF-MEK-dependent oxidative cell death involving voltage-dependent anion channels. *Nature* 447 864–868. 10.1038/nature05859 17568748 PMC3047570

[B184] Yamada K. Kaneko H. Shimizu H. Suzumura A. Namba R. Takayama K. (2020). Lamivudine inhibits Alu RNA-induced retinal pigment epithelium degeneration via anti-inflammatory and anti-senescence activities. *Transl. Vis. Sci. Technol.* 9 1–1. 10.1167/tvst.9.8.1 PMC7422901 32855848

[B185] Yang P. Peairs J. J. Tano R. Zhang N. Tyrell J. Jaffe G. J. (2007). Caspase-8-mediated apoptosis in human RPE cells. *Invest. Ophthalmol. Vis. Sci.* 48 3341–3349. 10.1167/iovs.06-1340 17591907

[B186] Yang W. S. Stockwell B. R. (2008). Synthetic lethal screening identifies compounds activating iron-dependent, nonapoptotic cell death in oncogenic-RAS-harboring cancer cells. *Chem. Biol.* 15 234–245. 10.1016/j.chembiol.2008.02.010 18355723 PMC2683762

[B187] Ye Z. He S. Z. Li Z. H. (2018). Effect of Abeta protein on inhibiting proliferation and promoting apoptosis of retinal pigment epithelial cells. *Int. J. Ophthalmol.* 11 929–934. 10.18240/ijo.2018.06.06 29977803 PMC6010384

[B188] Yin H. Xu L. Porter N. A. (2011). Free radical lipid peroxidation: mechanisms and analysis. *Chem. Rev.* 111 5944–5972. 10.1021/cr200084z 21861450

[B189] Young R. W. Droz B. (1968). The renewal of protein in retinal rods and cones. *J. Cell Biol.* 39 169–184. 10.1083/jcb.39.1.169 5692679 PMC2107515

[B190] Zhao H. Wang R. Ye M. Zhang L. (2019). Genipin protects against H2O2-induced oxidative damage in retinal pigment epithelial cells by promoting Nrf2 signaling. *Int. J. Mol. Med.* 43 936–944. 10.3892/ijmm.2018.4027 30569096 PMC6317649

